# Genetically-programmed, mesenchymal stromal cell-laden & mechanically strong 3D bioprinted scaffolds for bone repair

**DOI:** 10.1016/j.jconrel.2020.06.035

**Published:** 2020-09-10

**Authors:** Hosam Al-Deen M. Abu Awwad, Lalitha Thiagarajan, Janos M. Kanczler, Mahetab H. Amer, Gordon Bruce, Stuart Lanham, Robin M.H. Rumney, Richard O.C. Oreffo, James E. Dixon

**Affiliations:** a Regenerative Medicine & Cellular Therapies Division, The University of Nottingham Biodiscovery Institute (BDI), School of Pharmacy, University of Nottingham, Nottingham NG7 2RD, UK; b Bone and Joint Research Group, Centre for Human Development, Stem Cells and Regeneration, Human Development and Health, Institute of Developmental Sciences, Faculty of Medicine, University of Southampton, Southampton SO16 6YD, UK; c School of Pharmacy and Biomedical Sciences, University of Portsmouth, St Michael's Building, White Swan Road, Portsmouth PO1 2DT, UK

**Keywords:** Intracellular transduction, Controlled release, GAG-binding enhanced transduction (GET), PLGA, 3D bioprinting

## Abstract

Additive manufacturing processes used to create regenerative bone tissue engineered implants are not biocompatible, thereby restricting direct use with stem cells and usually require cell seeding post-fabrication. Combined delivery of stem cells with the controlled release of osteogenic factors, within a mechanically-strong biomaterial combined during manufacturing would replace injectable defect fillers (cements) and allow personalized implants to be rapidly prototyped by 3D bioprinting.

Through the use of direct genetic programming *via* the sustained release of an exogenously delivered transcription factor *RUNX2* (delivered as recombinant GET-RUNX2 protein) encapsulated in PLGA microparticles (MPs)*,* we demonstrate that human mesenchymal stromal (stem) cells (hMSCs) can be directly fabricated into a thermo-sintered 3D bioprintable material and achieve effective osteogenic differentiation. Importantly we observed osteogenic programming of gene expression by released GET-RUNX2 (8.2-, 3.3- and 3.9-fold increases in *OSX, RUNX2* and *OPN* expression, respectively) and calcification (von Kossa staining) in our scaffolds. The developed biodegradable PLGA/PEG paste formulation augments high-density bone development in a defect model (~2.4-fold increase in high density bone volume) and can be used to rapidly prototype clinically-sized hMSC-laden implants within minutes using mild, cytocompatible extrusion bioprinting.

The ability to create mechanically strong 'cancellous bone-like’ printable implants for tissue repair that contain stem cells and controlled-release of programming factors is innovative, and will facilitate the development of novel localized delivery approaches to direct cellular behaviour for many regenerative medicine applications including those for personalized bone repair.

## Highlights

• Efficient osteogenic programming of hMSCs by cell-transducing GET-RUNX2.
• Osteogenesis by controlled release of GET-RUNX2 from PLGA MPs.
• Mechanically strong biodegradable PLGA/PEG scaffolds containing MPs & hMSCs.
• 3D bioprinting of cell- and MP-laden scaffolds for personalized implants.

## Graphical abstract

**Figure:**
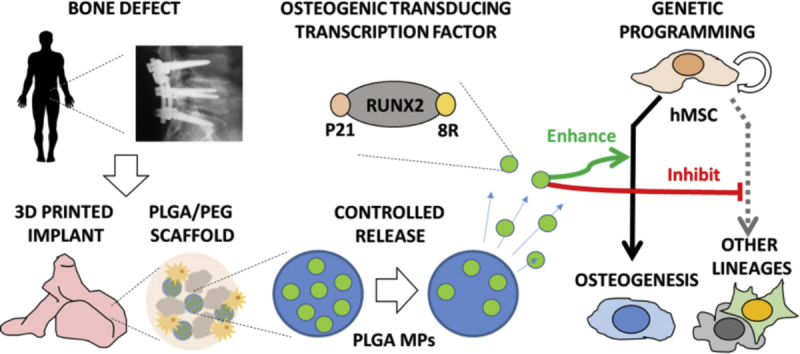

## 1 Introduction

Additive manufacturing of custom, defect-matched implants for regenerative medicine has significantly developed over the last decade [1]. For bone tissue engineering these approaches employ ceramic and polymeric materials to enable bone-like mechanically strong scaffolds to be generated, however the manufacturing process may not be bio-compatible (using high-temperatures, UV-light or organic solvents) [2]. As such only acellular implants are created and the incorporation of stem cells or therapeutic molecules can only occur post-fabrication. This prevents uniform seeding, patterning of cells or molecules to produce functionally complex tissues and long-term delivery is inhibited; thus bio-inductive strategies are often not effective or have less controlled activity [1]. Development of production processes which can be used intraoperatively; with cells and biomolecules directly included in the process is therefore vital for their clinical use.

Successful regenerative medicine strategies often rely on exquisite control of the biological microenvironment and supplementation of extrinsic therapeutic molecules to orchestrate cellular behaviour. Cellular processes like migration, proliferation, adhesion and differentiation occur in response to chemical cues present within the microenvironment such as interaction with extracellular matrix (ECM) and stimulation with morphogens or growth factors (GFs) [3,4]. These macromolecules trigger intracellular signal transduction pathways, with complex cross-talk between signalling cascades and eventual programming of cell behaviour through transcription factors (TFs) [[4], [5], [6]]. Directed cell differentiation can be achieved through the use of chemical induction or exogenous GFs or hormones, which might have non-specific pleiotropic effects. Therefore, there is a need for more efficient and targeted molecules that can be used in regenerative medicine. TFs can provide specific regulatory effect, which is considered ideal for cell fate; however, these TFs are difficult to deliver intracellularly.

Glycosaminoglycan-binding Enhanced Transduction (GET) peptides [[7], [8], [9], [10], [11], [12], [13], [14]] are delivery peptides that enhance transduction and endosomal escape of cargoes in cells. GET peptides contain Heparan Sulphate (HS) binding motifs that enhance binding to cell surface glycosaminoglycans (GAGs) which concentrate the cargo on cell membranes, combined with cell penetrating peptide (CPP) and endosomal escaping peptide elements to mediate uptake and escape, respectively. We have previously published the enhanced delivery characteristics and versatility of GET peptides, used as synthesized L-amino acid peptides or recombinant protein fusions to deliver recombinant proteins [9] [13], nucleic acids (pDNA, mRNA, siRNA) [7,11,12] and magnetic nanoparticles [14].

Our recent work has shown that recombinant TFs can be expressed, purified and delivered efficiently to control cell behaviour for various applications [7]. This system can be utilized to regenerate tissues if these molecules are supplied at functional dose, duration and location. Osteogenic TF RUNX2 (also called Core-binding factor alpha, CBFa1) is essential for osteoblast commitment, differentiation, matrix production, and mineralization during bone formation [15]. RUNX2 regulates downstream genes that determine the osteoblast phenotype and controls the expression of osteogenic marker genes such as *ALP, OP, OSX, COL1A1* (*type-I collagen), BSP*, and *OCN* [[16], [17], [18], [19]] in response to physiological signals [20].

We previously showed that GET-RUNX2 can be used to direct human Mesenchymal Stromal Cells (hMSCs) towards osteogenesis, removing the need to use pleiotropic compounds (such as dexamethasone), or GFs (such as BMP2) which may trigger unwanted off-target cellular responses. However, this TF needs to be supplied at a specific dose over a period of time for osteogenic induction [9].

Importantly, we have also shown the utility of GET peptides in regenerative medicine by delivering TFs RUNX2 and MYOD for osteogenesis and zonal myogenesis in three-dimensional gradients [9,21], respectively. Moreover, GET peptides have been used to enhance the delivery and transfection of nucleic acids for lung gene therapy and bone regeneration *in vivo* [11,12]. The latter delivering GF genes to enhance the repair of a critical size calvarial bone defect in rats [12].

Controlled and localized release of therapeutic molecules is one of the main factors that affect tissue regeneration within a scaffold [22]. The combination of biomaterials (scaffolds component), cells and therapeutic molecules can be used for localized and targeted regeneration therapies [23]. Poly-(DL-lactic acid-*co*-glycolic acid) (PLGA), which has been extensively studied as a degradable implant material [24], was used as a scaffold to study bone regeneration given its mechanical properties and enhanced mineralized tissue formation from its osteoconductive properties [25]. It has been shown that mixing PLGA with a plasticizer (such as Poly(ethylene glycol); PEG) will produce temperature-sensitive particles with a reduced glass transition temperature (*T*g) of 37 °C [26]. When these particles are mixed with an aqueous carrier solution at room temperature an extrudable paste is formed that can be moulded into any size or shape. Incubation of these pastes at 37 °C, surrounded by aqueous fluid leads to thermoresponsive liquid sintering and the formation of porous solid constructs [[21], [22], [23], [24], [25], [26]]. These constructs can be engineered with mechanical properties which are comparable to those of human cancellous bone [[21], [22], [23], [24],[27], [28], [29], [30]]. Previous studies utilizing this material have shown that proteins [23,25,26,27] and cells [21,23, 27] can tolerate the mixing and extrusion processes while maintaining activity and viability, respectively. Encapsulated proteins can be delivered over several weeks [26] and the microporosity inherent to these constructs, may also have beneficial effects on tissue ingrowth and angiogenesis *in vivo*.

Our recent study demonstrated that GET-tagged reporter peptides (mRFP, red fluorescent protein) could be efficiently encapsulated and its release controlled in PLGA microparticles (MPs) [13]. Moreover, this delivery system could provide sustained release of GET peptides for at least 7 days, without loss of transduction activity. In the current study, we hypothesized that these MPs could be employed to provide a sustained release system for TFs needed to promote osteogenesis of hMSCs. Herein, we have utilized biomaterials, cells and a therapeutic recombinant molecule to demonstrate that a combined system can be rapidly fabricated into mechanically strong osteogenic scaffolds or implants. GET-RUNX2 (RUNX2) loaded MPs, mixed with modified PLGA/PEG paste formulations was developed to provide a controlled release delivery of an osteogenic TF, a compatible microenvironment for the stem cells to generate new bone, and a system to deliver a mechanically strong implantable material, both *in vitro* and *in vivo*.

In this study, we aimed to demonstrate the efficient encapsulation, and release of active GET-RUNX2 protein from PLGA MPs which could be combined in a mechanically strong, 3D printable material. An innovative combined approach could have significant impact on clinical application of regenerative medicine in bone repair. Importantly, incorporation of viable hMSCs and a cell-fate programming system into a bioprinting process to produce osteogenic living implants could be demonstrated. The combination of this complete tractable system is the novelty and significance of our system.

## 2 Materials and methods

### 2.1 Protein synthesis and FITC tagging

CPP (eight arginine, termed 8R) and heparin binding domain (HBD, termed P21), which compose the Glycosaminoglycan – binding enhanced transduction (GET) system were used to tag the human *RUNX2* ORF to allow production of P21-RUNX2-8R protein [9]. cDNA constructs containing 8R, RUNX2 and P21 sequences were synthesized *de novo* (Eurofins MWG Operon, Ebersberg, Germany) and cloned into pGEX6-P1 expression vector (Novagen Watford, U.K.) [9]. Recombinant protein was expressed and purified as previously described in [28]. For protein tracking, P21-RUNX2-8R was tagged with FITC using NHS-Fluorescein as per manufacturer's instructions (Thermo Scientific) at 1:50 protein: label molar ratio and purified/buffer exchanged to PBS using Bio-Spin P-6 spin columns (Bio-Rad, Watford, UK).

### 2.2 PLGA microparticle fabrication

Poly (D,l-lactide-*co*-glycolide, (PLGA) LA:GA ratio: 50:50; Mw 52 kDa, Evonik Industries, USA) MPs were formed using solid-in-oil-in-water (S/O/W) emulsion as previously described [13,29]. Briefly, 1 mg of P21-RUNX2-8R, was mixed with 0.5 mg PEG 6000 (Sigma-Aldrich, UK) and 0.25 mg L-Histidine (Sigma-Aldrich, UK) and freeze dried overnight. Blank MPs were prepared by freeze drying 0.5 mg PEG 6000 only. PLGA was dissolved in dichloromethane (DCM, Fisher, UK) (200 mg of PLGA was dissolved in 1.5 ml DCM) and added to the freeze dried powder. The theoretical GET-RUNX2 loading was 1 mg in 200 mg PLGA (0.5%) The solution was then mixed by vortexing. Two hundred microliters of phosphate buffered saline (PBS, Gibco, UK) was added to the solution and vortexed to produce a homogenous emulsion. Four millilitres of 0.25% (*w*/*v*) methylcellulose (Sigma-Aldrich, UK) solution was then added to the mixture and vortexed again. The resultant emulsion was then poured into 400 ml of distilled water; and the resultant (S/O/W) emulsion was left stirring for 3 h to allow DCM evaporation. The hardened MPs were then collected by centrifugation and washed three times with distilled water. The obtained MPs were then freeze dried (Edwards Modulyo D IMA Edwards, UK) for 24 h.

### 2.3 Extrusion bioprinting

A RegenHU 3D Discovery system (Switzerland) was used for 3D printing with Huber Pilot ONE temperature controller (Switzerland). PLGA/PEG particles (≤50 μm) were mixed by spatula with different carriers, cells and microparticles at 4 °C before being transferred into a sterile syringe. Mixing was performed until a homogenous colour (of the phenol-red cell culture media) was achieved as previously [30]. Syringes were placed into the printer mount and allowed to equilibrate with the printing temperature (25 °C). During printing, the pressure was manually adjusted (1–3 bar) to ensure adequate flow of material through the 20 gauge (0.61 mm) tapered syringe tip (Adhesive dispensing Ltd. UK). The print speed varied between 20 and 60 mm/s. BioCAD software (RegenHU) was used to design the lattice structure to be printed. Dissection microscope Nikon SMZ1500 was used to image the printed structures. For 3D printing of the L5 vertebra, SLS files were downloaded from: http://www.thingiverse.com/thing:781206. For L5 vertebrate; PLGA/PEG with Pluronic F-127 (PF127) 18% (with 1% Tri-acetin) 1:1.5 ratio was mixed and loaded to the printer, the printing temperature was 25 °C.

### 2.4 Cell seeding pre-fabrication

Cells were mixed with the carrier (18% PF-127 with 1% Tri-acetin) with final density of 1 × 10^7^/ml and mixed with PLGA/PEG particles in 1:1.5 (PLGA/PEG:carrier, *w*/*v*) ratio at 4 °C as described previously [30]. The resultant mixture was then loaded to the mould or printing syringe and heated to 25 °C. The mixture was then printed, and sintered at 37 °C for 30 min. The sintered scaffolds were then washed in cold PBS for 45 min and then cultured in cell culture media.

### 2.5 Cell seeding post-fabrication

To ensure sterility, the scaffolds were treated with 1% antibiotic and antimycotic solution for 20 min at 37^0^C and washed extensively with sterile PBS as previously described [13,31]. Fifty thousand cells in a 20 μl suspension was pipetted onto each sintered scaffold. The scaffolds were incubated for 1 h at 37 °C, to allow attachment, prior to addition of 1 ml media to each well.

### 2.6 Osteogenesis assay

DMEM: F12 media (Life technologies, Paisley, UK) supplemented with 10% (*v*/v) fetal bovine serum, 2 mM l-glutamine, 100 units/ml penicillin and 100 μg/ml streptomycin (Sigma) was used as the basal media for osteogenic media. Fifty thousand cells per scaffold (containing blank MPs or P21-RUNX2-8R loaded MPs) were seeded and cultured for 4 weeks depending on the experiment with appropriate media. 50 μg/ml l-Ascorbic acid 2-phosphate sesquimagnesium salt hydrate (Sigma) and 10 mM β glycerophosphate disodium salt pentahydrate (Acros Organics) were added to the basal media for osteo-permissive medium (OP). To make osteo-inductive media (OI), 100 nM Dexamethasone (Sigma) was added to the osteo-permissive (OP) medium. Cells were cultured for 3–4 weeks for complete osteogenesis. Medium was changed every other day.

### 2.7 Cell viability assay

PrestoBlue® cell viability assays (Invitrogen Life Sciences, UK) were performed on day 3, 6 and 9 post-seeding on three scaffolds per time point. Each scaffold was submerged in 900 μl of media and 100 μl of PrestoBlue was added to each well and mixed. The scaffolds were then incubated at 37 °C for 2 h. Three 100 μl media samples were taken from each well and were read on Tecan plate reader with the excitation wavelength set to 560 nm and the emission wavelength set at 590 nm. Blank sample readings were subtracted and results were compared with corresponding standard curve of known cell numbers to calculate the number of cells per scaffold.

### 2.8 *In vivo* bone defect assay and μCT

hMSC populations were selected by magnetic separation (STRO-1+) from adherent mononuclear cell fractions from human bone marrow obtained during routine knee/hip replacement surgeries with full ethical approval and informed consent from the patients in accordance with approval from Southampton & South West Hampshire Local Research Ethics Committee, UK (Ref: 194/99/w). Briefly, bone marrow aspirate was thinned with basal media (DMEM supplemented with 10% (*v*/v) FBS, 1% (v/v) sodium pyruvate, 1% (v/v) non-essential amino acids, 1% (v/v) penicillin-streptomycin, 1% (v/v) l-glutamine, and layered onto Lymphoprep density gradient media (Stem Cell Technologies). Following centrifugation at 800 ×*g* for 40 min, the intermediate interface of mononuclear cells was removed and washed three times with media. These cells were then selected for the marker STRO-1 using an in-house STRO-1 antibody (original hybridoma courtesy of Dr. Beresford, University of Bath, UK) using a MACS kit from Miltenyi Biotech as previously detailed [32]. Only adherent STRO-1+ cells were cultured. Cells from two patients were used in two separate experiments. Scaffold containing P21-mRFP-8R or P21-RUNX2-8R MPs were cut into approximately 1 mm^3^ sized pieces and 1-3 × 10^4^ STRO-1+ hMSCs were added to each scaffold. Cells were incubated on the scaffold at 37 °C, 5% CO_2_ for 3–4 days. All *in vivo* studies were undertaken following approval from the local Animal Welfare and Ethics Review Board (AWERB) University of Southampton and carried out in accordance with the guidelines and regulations stipulated in the Animals (Scientific Procedures) Act, UK 1986 under the approved Home Office Project license (PPL 96B16FBD). All mice were raised within the University of Southampton Biomedical Research Facility and were housed in appropriate environments in rooms maintained at 22 ± 2 °C with a 12 h light: 12 h dark cycle. Eight week old male athymic nude BALB/c mice were used for the study with 4–6 animals per group per patient. A 1 mm drill-hole defect was made in the right distal femur, and then a single 1 mm^3^ scaffold piece with hMSCs was added to the hole. After surgery and at 2 weekly intervals, mice were anaesthetized using isoflurane inhalation. Femora were scanned using a Skyscan 1176 *in vivo* micro(μ)CT scanner (Bruker microCT, Kontich, Belgium). All scans were taken at 65 kV, 385 μA with 1 mm aluminium filter, and 0.7° rotation step. Individual 2D cross-sectional images were reconstructed using Bruker NRecon software version 1.6.10.2. Voxel resolution was 35 μm. All reconstructed bones were set to the same orientation with the transverse plane perpendicular to the long axis of the bone using Bruker Dataviewer software. For each animal, the scan at the time of surgery (week 0) was used as a reference, and subsequent scans were aligned to this scan using 3D Registration in Dataviewer. A 1 mm diameter volume of interest centred on the defect hole at week 0, was used on all subsequent scans to determine bone volume and density at the defect site. The depth of the defect is the thickness of the cortical bone (approx. 250 μm). The images are semi-transparent and represent the bone density for the entire 3D reconstruction. High density bone was defined based on the intensity of signal measured by μCT as we have previously published [33] (lowest density bone set at 0.25 g/cm^3^).

### 2.9 Statistical analysis

Statistical analysis was carried out using GraphPad Prism (version 7). For ALP assays the statistical significance was determined using one-way ANOVA with Holm-Sidak *post hoc* test for ALP study. For gene expression analyses. Proliferation and mechanical testing data was analysed by two-way ANOVA and Tukey's test assuming confidence levels of 95% (*p* < .05). For mechanical testing the homogeneity of variances across experimental groups was determined by Levene's test. Appropriate *post hoc* procedures were used after one way ANOVA for pairwise comparison of experimental groups. In cases of equal variance Tukey's procedure was applied, whilst the Games-Howell procedure was used for cases of unequal variance. For the *in vivo* study, significance was determined using independent sample *t*-test in SPSS version 25 (IBM, Woking, UK). Results were considered significant at *p* < .05, mean values are given plus or minus the standard deviation (SD) or Standard error or the mean (SE).

## 3 Results

### 3.1 Efficient encapsulation and controlled release of GET-RUNX2

**Fig. 1:**
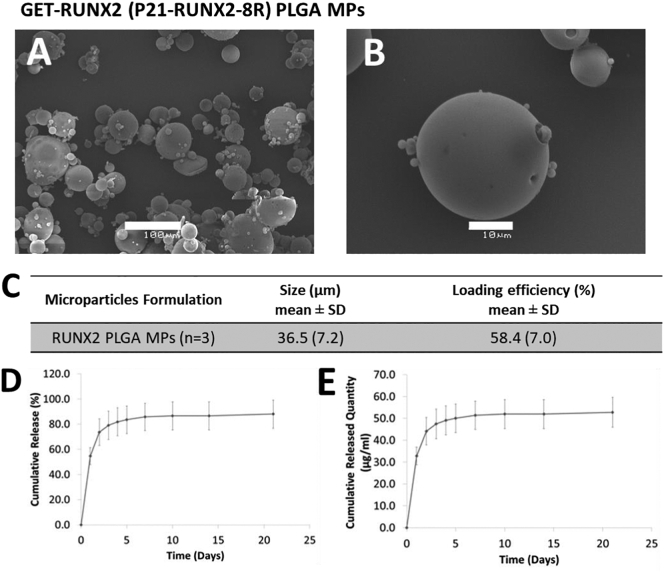
MP characterization and P21-RUNX2-8R-FITC in vitro release profile (pH 7.4 at 37 °C). MPs were fabricated using PLGA (50:50), loaded with P21-RUNX2-8RFITC (0.5% (w/w)), mixed with PBS 0.1% (w/w) and L-Histidine 25% (w/w). (A, B) SEM analyses for MPs (scale bars represent (A) 100μm and (B) 10 μm). (C) Summary of size characterization and corresponding loading efficiencies (n = 3). (D, E) Protein release quantified using Micro BCA assay kit. (D) Relative cumulative protein release. (E) Cumulative protein quantity (using same data as D). Error bars indicate SD (n = 3).

PLGA MPs were fabricated using an optimized S/O/W double emulsion process and encapsulated GET-tagged RUNX2 protein (P21-RUNX2-8R, termed GET-RUNX2). PBS was added to the formulation to increase porosity thereby accelerating the protein release [13]. PBS acts as a porogen, which is expected to increase MP porosity and generate channels within and on the MP surface. Porous MPs were needed to accelerate the GET-RUNX2 release as previously shown [13]. MPs prepared using the S/O/W methodology showed spherical structure with smooth and porous surface due to the addition of PBS (Fig. 1A&B). Porosity of the fabricated PLGA MPs was assessed visually using SEM images and these were similar to those in our previous work [13]. The mean diameter of the MPs was 36.5 ± 7.2 μm, with average loading efficiency of 58.4 ± 7.0% (generating 2.9 ± 0.2 μg per mg of PLGA MPs) (Fig. 1C & S1). P21-RUNX2-8R MP dose was optimized to obtain release quantities matching or greater than the dose needed (60 μg GET-RUNX2 during the first week) to initiate osteogenic differentiation of hMSCs [9]. The release profile showed comparable GET-RUNX2 quantities (*i.e.* 30 μg in the burst release phase and a further 30 μg over 5 days) (Fig. 1D&E). Moreover, this represented more than 90% of the protein loaded in MPs, which confirmed that minimal protein is released after the first week. This was important as RUNX2 activity is needed predominantly during the early stages of osteogenesis, and overexpression or prolonged activity of RUNX2 during later stages delays matrix mineralization [34].

### 3.2 PLGA released GET-RUNX2 retains intracellular transcriptional activity

**Fig. 2:**
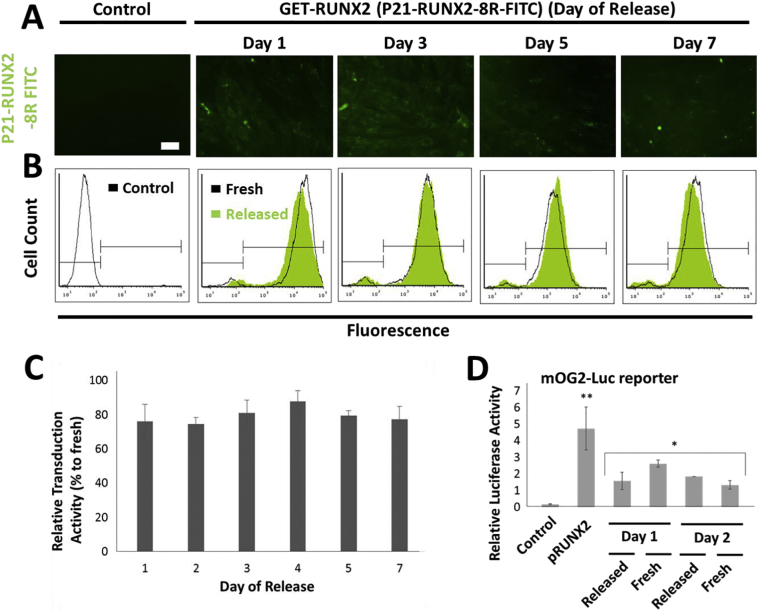
Retained transduction and transcriptional activity of released P21- RUNX2-8R. PLGA MPs were prepared using S/O/W method, loaded with P21- RUNX2-8R-FITC (0.5% (*w*/w)). (A) Transduction activity of the released protein was assessed in hMSC monolayers. Representative fluorescence microscopy images of the hMSCs treated for 24 h with the released P21-RUNX2-8R-FITC (Scale bar represents 100 μm) collected on the days indicated. (B) Flow cytometry histograms showing cellular uptake of P21-RUNX2-8R-FITC in hMSCs. The green histograms are the released P21-RUNX2-8R-FITC, while the black histograms are fresh experimental controls of P21-RUNX2-8R-FITC delivered at the same released concentrations (between 3 and 20 μg/ml)(the same fresh controls were used for day 5 and 7). Gates of from control cells showing negative (left) and positive (right) cells. (C) Relative transduction activity of released P21- RUNX2-8R-FITC compared to fresh protein. Values are the mean fluorescence intensity (MFI) per cell from flow cytometry. (D) Transcriptional activity of the released P21-RUNX2-8R-FITC measured using mOG2-Luc RUNX2-responsive luciferase reporter gene assay. pRUNX2 (plasmid DNA, pSIN-RUNX2) transfection was used as a positive control. Values induced after 1 or 2 days transduction are presented. Error bars indicate SD (*n* = 3). ** *p* < .05. (For interpretation of the references to colour in this figure legend, the reader is referred to the web version of this article.)

As GET-RUNX2 can be encapsulated and released in osteogenic-inductive quantities from fabricated PLGA MPs, it was crucial to demonstrate that it also retained its biological activity (both transducing into cells *via* the GET peptides and retained transcriptional activity). We previously demonstrated that PLGA MPs benefit from the inclusion of L-Histidine during fabrication to maintain the macropinocytotic-activity of GET peptides [13]. L-Histidine is needed to maintain complete activity of GET peptides upon increased acidity in the microenvironment within the PLGA MPs [13]. The transduction activity of GET-RUNX2 was confirmed by comparing released protein to non-encapsulated controls. To facilitate visualization, we labelled GET-RUNX2 with FITC (NHS-fluorescein). Released GET-RUNX2-FITC was collected over the 7 day time course and was incubated with plated hMSCs to assess internalization (Fig. 2A), transduction (Fig. 2B&C) and transcriptional activity (Fig. 2D). Importantly, we compared the released samples to non-encapsulated ‘fresh’ controls diluted to the same final concentration (*i.e.* the released protein was quantified and compared against fresh protein sample of the same concentration). Incubated cells were then evaluated for transduction by fluorescence microscopy and flow cytometry analyses (Fig. 2A&B). Fluorescent microscopy showed significant GET protein internalization for the collected samples released from PLGA MPs (Fig. 2A). Flow cytometry results showed high transduction activity with relative transduction activity not statistically different (~70% of ‘fresh’ GET-RUNX2 levels) for all the studied samples (Fig. 2B&C). Therefore it was clear that the released protein retained their transduction activity throughout at least a 7 day release period.

In order to study the transcriptional activity of the released GET-RUNX2, the immediate transcriptional activation of a RUNX2-responsive luciferase reporter containing the transcriptional responsive sequence from the *OSTEOCALCIN (OCN)* promoter (mOG2-Luc) was tested (Fig. 2D), as used previously [9]. The activity of the released GET-RUNX2 samples was compared with non-encapsulated ‘fresh’ GET-RUNX2. Cells were exposed to the released GET-RUNX2 before and after reporter transfection which generates the most pronounced transcriptional activity as previously shown [9]. We observed that the released GET-RUNX2 (collected at day 1 and 2) induced mOG2-Luc reporter expression to a magnitude comparable to the ‘fresh’ GET-RUNX2 (*i.e.* 1.57 (±0.51) fold verses 2.21 (±0.21) fold respectively). Transcriptional activity enhancement was not significant post-day 2 due to the smaller amounts of GET-RUNX2 released. Although this assay is not highly responsive, transcriptional enhancement was shown significant for immediate activity above control untreated levels. These findings clearly indicate that the released GET-RUNX2 retained its transduction and transcriptional activity upon releasing from PLGA MPs.

### 3.3 PLGA/PEG scaffolds support hMSC attachment and proliferation

In order to generate a delivery vehicle for cells and MPs, and capacity for localized delivery of GET-RUNX2, scaffolds were prepared aiming to incorporate the fabricated MPs with seeded cells on top or within them. The goal was to finally incorporate the full system with hMSCs and GET-RUNX2 MPs into a 3D bioprintable formulation.

**Fig. 3:**
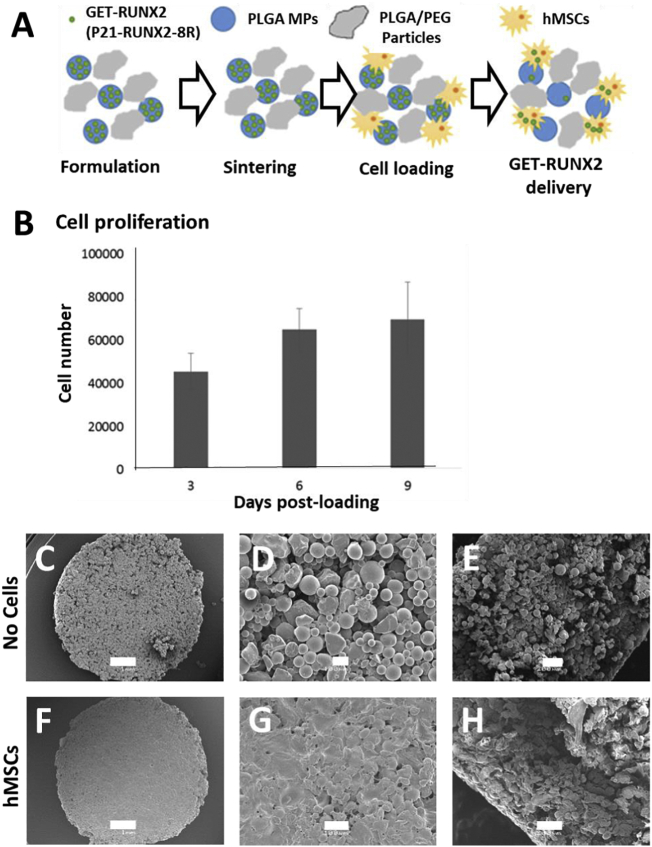
hMSCs attach and proliferate on PLGA/PEG scaffolds. (A) Schematic of scaffolds fabrication and cell attachment on scaffolds after sintering. (B) Number of viable hMSCs and proliferation measured by the Prestoblue metabolic activity on day 3, 6 and 9 post-seeding (50,000 cells/scaffold). (C-E) SEM images of scaffolds without cells (scale bar for C, D and E are 1 mm, 100 μm and 200 μm, respectively). (F-H) SEM images of cell-seeded scaffolds 3 days post-seeding (scale bar for F, G and H are 1 mm, 100 μm and 200 μm, respectively). E and H images are of cut scaffolds showing internal morphology.

Our previously developed temperature sensitive scaffold (composed of PLGA/PEG) was used [13]. This system allows incorporation of PLGA MPs with the PLGA/PEG scaffold particles that will sinter to form a hard porous scaffold at 37 °C, whereas, at room temperature the mixture remains as a paste. PLGA/PEG temperature sensitive particles were prepared as previously detailed [13] (Fig. 3A). GET-RUNX2 loaded MPs were mixed with PBS and the PLGA/PEG material before sintering, the material was then loaded into PTFE moulds creating cylindrical scaffolds (3 mm depth x 6 mm diameter) and sintered for 2 h at 37 °C. Blank MPs were used for non-loaded scaffold controls. Initially, hMSCs were seeded on these sintered scaffolds and cultured for further studies. PrestoBlue assay was performed on days 3, 6 & 9 to confirm cell metabolism and therefore viability on scaffolds. The results demonstrated that the scaffold is conducive for cell proliferation (*i.e.* cell number increased from around 40,000 at day 3 to around 65,000 at day 9) (Fig. 3B). SEM images showed a different surface morphology for cell-seeded scaffolds (compared to non-seeded scaffold) with cells covering the scaffolds with no vacant pores on the surface (Fig. 3C–H). This was similar to previous analyses of cell attachment and growth on PLGA/PEG scaffolds [26].

### 3.4 hMSCs differentiate in response to GET-RUNX2 released from PLGA MPs

For hMSC osteogenic differentiation *in vitro*, ascorbic acid and β-glycerophosphate are essential for final stages of bone nodule formation by promoting collagen matrix production and providing inorganic phosphate for mineralization, respectively. In order to test the effect of GET-RUNX2 on bone differentiation, media containing these additives (termed ‘osteo-permissive’, OP) was used during differentiation studies. hMSCs were seeded on scaffolds containing either GET-RUNX2-loaded MPs or blank MPs. Osteo-inductive media (OI) containing 100 nM Dexamethasone (along with 50 μg/ml ascorbic acid and 10 mM β-glycerophosphate) was used as a positive control on scaffolds containing blank MPs. A full assessment of osteogenic differentiation was carried out at different stages using Alkaline phosphatase (ALP) quantification and quantitative gene expression analyses (Q RT-PCR) at week 1, OCN staining and Q RT-PCR at week 2, and von Kossa staining at week 3.

**Fig. 4:**
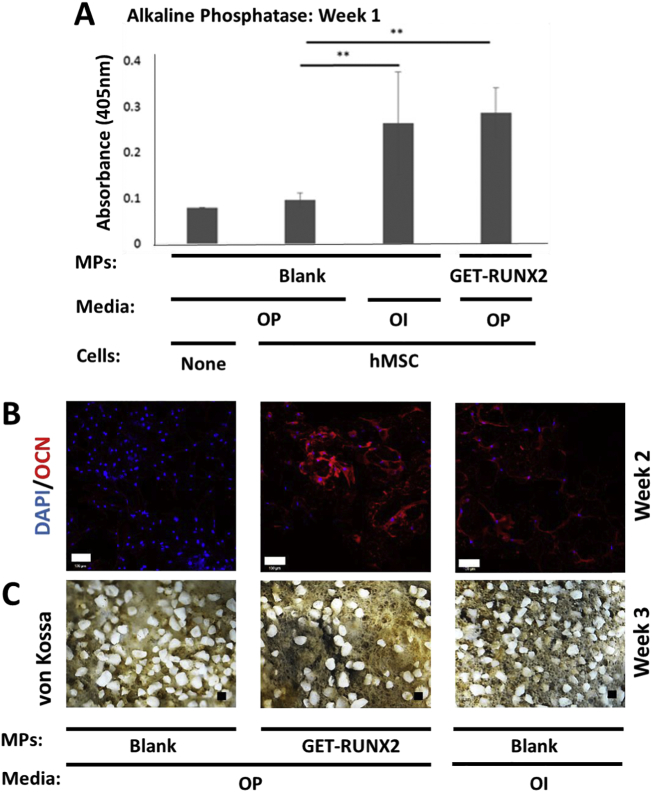
hMSC osteogenic differentiation on PLGA/PEG scaffolds. Differentiation markers for hMSCs seeded on scaffolds in osteo-permissive (OP) and osteo-inductive media (OI). (A) Alkaline phosphatase (ALP) quantification at week one comparing blank and GET-RUNX2 MPs, with OI conditions and without hMSCs as a control. (B) OSTEOCALCIN (OCN) immunostaining (red) with DAPI nuclear staining (blue) at week 2 (scale bar is 100 μm). (C) Von kossa-stained samples at week 3 (scale bar is 100 μm). Blank MPs represents cell seeded scaffold containing blank MPs cultured in osteo-permissive (OP) media. Loaded MPs represents cell-seeded scaffolds containing P21-RUNX2-8R-loaded MPs cultured in osteo-permissive (OP) media. OI media represents cell seeded scaffolds containing blank MPs cultured in osteoinductive media (OI). ** is *P* < .01.

ALP assays were performed on three types of scaffolds; blank scaffolds in OP media, blank scaffolds in OI media and GET-RUNX2 (RUNX) loaded scaffolds in OP media. ALP levels, as measured by absorbance at 405 nm, were comparable between GET-RUNX2 loaded scaffolds and the scaffolds in OI media and significantly higher than the blank scaffolds cultured in OP media (Fig. 4A).

OSTEOCALCIN (OCN) is a bone-specific protein synthesized by osteoblasts and its expression confirms full osteogenic commitment before progression towards bone nodule formation [[35], [36], [37]]. OCN staining was therefore conducted at day 14 post-fabrication (Fig. 4B). Expression of OCN was visually comparable to scaffolds cultured in OI media and those containing GET-RUNX2 loaded MPs. On the other hand, there was minimal OCN expression observed in blank scaffolds seeded in OP media (Fig. 4B). Mineralization was assessed using von Kossa staining at day 21 (Fig. 4C). Bone nodules (black material) were visible in scaffolds seeded in OI media and scaffolds containing P21-RUNX2-8R loaded MPs. All tested scaffolds showed the white composite PLGA scaffold, which was less evident in more differentiated conditions (with GET-RUNX2 MPs or with OI) due to being coated with black von Kossa stained mineral deposits.

**Fig. 5:**
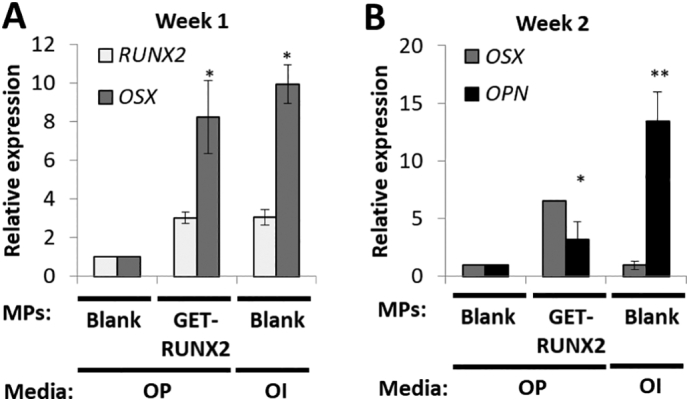
hMSC osteogenic gene expression analysis on PLGA/PEG scaffolds. Blank MPs represents cell-seeded scaffold containing blank MPs, cultured in osteo-permissive (OP) media. RUNX2-loaded MPs represents cellseeded scaffolds containing P21-RUNX2-8R loaded MPs, cultured in osteopermissive (OP) media. OI represents cell-seeded scaffolds containing blank MPs, cultured in osteo-inductive (OI) media. RNA was extracted from hMSCs at week 1 (A) and week 2 (B) and gene expression analysis using Q-RTPCR was performed. The results were plotted based on expression fold change to noninduced hMSCs on blank scaffold using the ΔΔCT method. Data is presented as mean ± SE. * p.

Finally, we studied the molecular effects of GET-RUNX2 loaded MPs on early and late osteogenic gene induction in hMSCs by Q RT-PCR (Fig. 5). Significant activation of osteogenic genes could be observed in GET-RUNX2 loaded cells in both week 1 and 2. As expected, GET-RUNX2 loaded MPs released active P21-RUNX2-8R which induced endogenous *RUNX2* expression in hMSCs after week 1 (Fig. 5A). *OSTERIX (OSX)*, one of the prime target genes for RUNX2 is significantly activated (8.2 fold over non-induced control; *p* < .05) in week 1 of GET-RUNX2 loaded hMSCs (Fig. 5A). Expression of these two genes in GET-RUNX2-loaded MP cells was similar to cells cultured on scaffolds in OI medium (positive control). *OSTEOPONTIN (OPN)*, a bone matrix protein [38] however was lower in GET-RUNX2-loaded MP cells in comparison to positive control cells at week 2 (Fig. 5B). This may be due to the continuing release of RUNX2 which would be hypothesized to delay terminal differentiation of the hMSCs in comparison to the OI positive control.

### 3.5 hMSCs colonize the surface of PLGA/PEG scaffolds

We conducted SEM evaluation of scaffolds at day 21. Scaffolds without cells (incubated in media for 21 days) were tested to evaluate polymer degradation. The cell-seeded scaffolds showed less porosity as the cells completely covered the surface as we have observed previously [26]. Cells were seen to only grow significantly on the surface (as shown in the scaffolds cut in half, Fig. S2C,F,I,L) which can be attributed to poor migration into scaffold pores as well as the lower porosity of the prepared scaffolds. The media used (OP or OI) did not have an overt impact on cell morphology, as in both cases the cells covered the scaffolds with no noticeable difference (Fig. S2). The microstructure evaluation of scaffolds (using micro (μ)CT) showed comparable results between all scaffolds (Fig. S3). The cell distribution was mainly on the surface of the scaffolds, with little cells within the scaffold structure, as seen in SEM images of scaffold cross section (Fig. S2). This was similar to previous extensive studies with cells on this material [26]. Specifically scaffolds that had the highest osteogenic differentiation; those with GET-RUNX2 MPs (Fig. S3C) and those cultured in OI media (Fig. S3D) had lower internal numbers of hMSCs (density of blue signal). This may be expected as differentiating hMSCs will have, typically, lower proliferation rates.

### 3.6 PLGA/PEG particles can be 3D printed by addition of Pluronic F127

Pluronic(P) F127 is a temperature-sensitive FDA approved polaxmer [39], formulated as a triblock copolymer of poly (ethyleneoxide)/poly (propyleneoxide)/poly (ethyleneoxide) [[39], [40], [41]]. PF127 has thermo-reversible gelation properties and has been widely used as cell carrier and for various 3D printing applications [[39], [40], [41], [42]]. At certain concentrations, aqueous PF127 solutions gel at physiological temperatures [39]. In order to develop a printable formulation of our system, the material would have to possess a certain level of viscosity [43,44] that allows continuous flow of the material through an extrusion printing head/needle. Previously this material has been successfully combined with several carriers to allow 3D printing, and sintering in the presence of proteins and cells [27]. In order to achieve the needed viscosity, we tested the use of PF127, pre-mixed before sintering with the PLGA/PEG particles. PLGA/PEG successfully sintered in our groups previous studies [30]. No MPs were included at this stage to allow the optimization of an extrudable formulation and printing process, once the parameters had been optimized, the MPs were included into the system. Viscosity for various concentrations of PF127 at different temperatures was studied. It was concluded that suitable PF127 concentration for printing should be between 15 and 20% (*w*/*v*). The temperature was fixed at 25 °C using a heated print head with PF127 being a gel at this temperature and with sheer thinning suitable for printing (Fig. S4 shows concentrations and ratios for printing and example tests). From our analyses it was clear that the most suitable printing concentration was 18% (w/v) PF127 added in 1:1.5 ratio (PLGA/PEG particles:PF127) which allowed extrusion and PLGA/PEG sintering. There was no appreciable change in mechanical properties with handling of the sintered PLGA/PEG scaffold with addition of PF127 at these concentrations. PF127-containing scaffolds displayed compressive strength (0.88 ± 0.06 MPa) (Fig. S5A) and elastic (Young's) modulus (7.87 ± 0.01 MPa) (Fig. S5B), similar to previously published (0.92 ± 0.05 and 8.76 ± 0.05) with four hours sintering [30]. A further modification was developed to accelerate the sintering of printed scaffolds containing PF127. Tri-acetin (1%) was added to the PF127 solution; Tri-acetin is a plasticizer [45,46] and reduces the time needed to stabilize scaffolds before media is added (from 2 h down to 30mins to remain intact). Addition of Tri-acetin to PF127-containing scaffolds accelerated compressive strength (0.56 ± 0.09 verses 0.29 ± 0.03 MPa with and without, respectively at 30mins) (Fig. S5A) and elastic (Young's) modulus (2.76 ± 0.03 verses 1.04 ± 0.04 MPa with and without, respectively at 30mins) (Fig. S5B) [30]. Importantly, although these values are lower than those obtained in other bone tissue engineering studies, with harsher fabrication, they are nonetheless within the normal ranges for cancellous bone and the tissue is thus, mechanically strong [[21], [22], [23], [24],[27], [28], [29], [30]].

### 3.7 Scaffold integrity is retained with Pluronic F127 which can be removed rapidly with low temperature washing

At a temperature of 37 °C, PF127 forms an unstable gel which persists even when immersed in culture media for extended periods [39] and can affect cell viability [40]. Therefore, we hypothesized that PF127 would be best removed, and possibly this could be achievable by diluting it away at lower temperatures.

Accordingly, scaffolds were washed in PBS at 4 °C for up to 45 min after sintering and placed at 37 °C for 30 min. As a control to evaluate the washing efficiency, scaffolds were also washed in PBS at 37 °C. It was thought that if the washing process was not efficient at 4 °C, the remaining PF127 would gel at 37 °C and the scaffolds would swell. The scaffolds washed at 4 °C for over 10mins in cold PBS showed smooth structure with no swelling (Fig. S7), on the other hand, scaffolds washed at 37 °C showed swollen structure with PF127 adsorbed on the surface and the gel retained (Fig. S7). Mechanical testing of PF127-containing scaffolds (with Tri-acetin) with and without cold PBS washes showed minimal effect on both compressive (1.47 ± 0.08 verses 1.58 ± 0.07 MPa with and without, respectively at 4 h) (Fig. S6A) and elastic (Young's) modulus of scaffolds(10.85 ± 0.26 verses 11.23 ± 0.12 MPa with and without, respectively at 4 h) (Fig. S6B). Mechanical strength within the range of cancellous bone [[21], [22], [23], [24],[27], [28], [29], [30]] was therefore retained following PF127 removal.

### 3.8 hMSCs proliferate on scaffolds after removal of Pluronic F127

After optimizing the formulation for printing with the addition and removal of PF127, it was important to determine the cell density optimal for printing, viability and differentiation, and to study the effect of the whole process on cell viability and proliferation. Accordingly, various cell densities were studied and cell proliferation was tested. The scaffolds were prepared by mixing immortalized hMSCs (ihMSCs) with PLGA/PEG particles (using 18% PF127 with 1% Tri-acetin at 1:1.5 ratio (PLGA/PEG:carrier) formulation). The tested cell densities (0, 0.05, 0.1, 0.25, 0.5 and 1 × 10^6^ per scaffold) were mixed, and the paste was loaded into PTFE moulds and sintered at 37 °C. The scaffolds were then washed in PBS at 4 °C for 45 min. Scaffold disintegration was evaluated before and after washing by microscopy (Fig. S8A). The results showed that intact sintered scaffolds (scaffolds were sintered for 30 min at 37 °C) remained intact after washing in PBS at 4 °C (Fig. S8A), indicating that the sintering time was enough for the scaffolds to maintain the structure and that PF127 removal did not significantly affect the scaffold structure nor integrity. This mirrored the mechanical testing previously demonstrating strong scaffold generation (Fig. S6 & S7) [30]. Scaffolds were cultured in growth media at 37 °C, with PrestoBlue cell metabolism assays performed to assess cell survival and proliferation (Fig. S8B). Increased initial cell concentrations resulted in enhanced cell retention in the scaffolds during washing and more robust attachment and proliferation over the 10 day assessment (Fig. S8B). Any cell number > 0.1 × 10^6^ cells/scaffold showed effective proliferation, with 1 × 10^6^ cells likely to have saturated the scaffolds at day 5–7 (with a plateau in metabolic activity). This demonstrated that addition of PF127 and Tri-actin, sintering, washing and subsequent culture was compatible with ihMSC viability and proliferation.

### 3.9 Successful cytocompatible bioprinting of hMSC-laden PLGA/PEG scaffolds

**Fig. 6:**
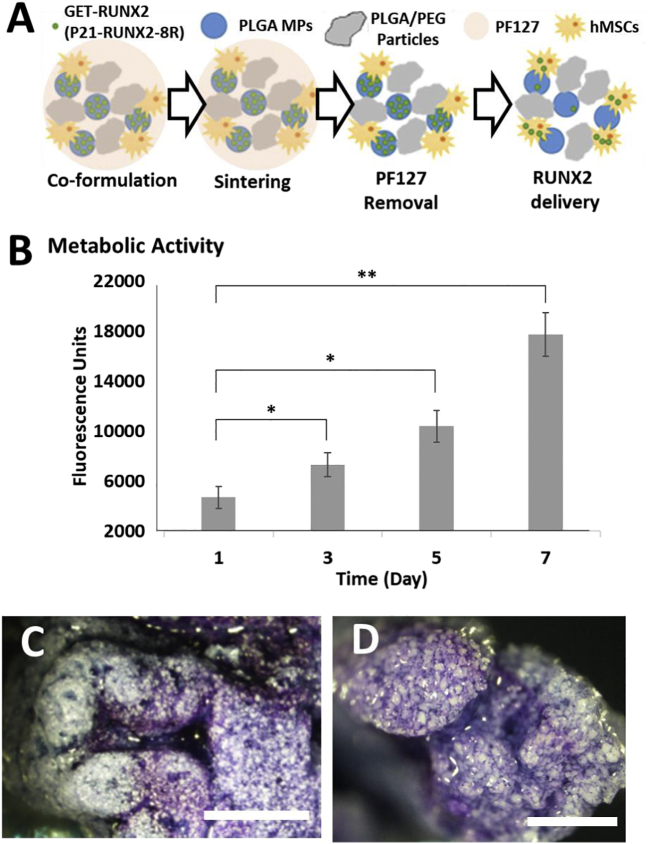
hMSCs attach and proliferate within 3D bioprinted PLGA/PEG scaffolds. (A) Schematic of hMSC-laden scaffold fabrication with PF127, sintering and PF127 removal after sintering. PLGA/PEG particles were mixed with 1% Tri-actin and Pluronic 18% solution in 1:1.5 ratio. The solution was mixed with hMSCs (1 × 107 per ml). The mixture was loaded to the printing syringe and printed at 25 °C. After sintering at 37 °C and washing in cold PBS at 4 °C, scaffolds were then cultured in media for 7 days. (B) Cell proliferation was evaluated by PrestoBlue assays at different time points for the cultured scaffolds. Error bars indicate SD (*n* = 3). (C, D) Toluidine blue stain for cells attached to the scaffold at day 7. (C) Cells at the scaffold surface, and (D) internal staining by breaking a scaffold strand. Scale bar is 1 mm. Data is presented as mean ± SD. * *p* < .01.

**Fig. 7:**
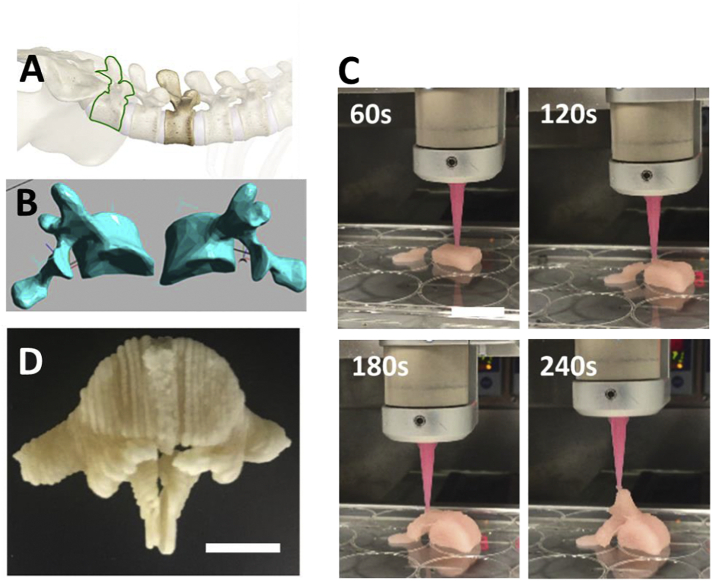
3D bioprinting of an L5 human adult lumber vertebra. (A) L5 human adult vertebra (25 mm height, 50 mm width) (highlighted in green) was chosen to demonstrate rapid 3D bioprinting of hMSC-laden PLGA/PEG scaffold as a personalized implant. (B) STL file of the L5 vertebra was divided into two halves for 3D bioprinting. (C) Half L5 vertebrae were fabricated in less an 4 min (240 s). (D) L5 vertebrae halves could be bonded with further PLGA/PEG paste (PLGA/PEG mixed with 1% Tri-acetin and 18% PF127 (1:1.5 ratio) and ihMSCs), sintered and cold washed to generate an hMSC-laden personalized scaffold. Scale bars are 20 mm. (For interpretation of the references to colour in this figure legend, the reader is referred to the web version of this article.)

The final aim of our studies was to fully confirm that the developed system was a 3D bioprintable scaffold able to be co-printed with viable stem cells and provide an osteopermissive hard yet porous structure for bone regeneration applications. As a full demonstration, ihMSCs were added to carrier (18% PF127 with 1% Tri-acetin) with final density of 1 × 10^7^/ml (equivalent to 0.5 × 10^6^/moulded scaffold) and mixed with PLGA/PEG particles in 1:1.5 ratio at 4 °C. The mixture was then loaded to the printing syringe and heated to 25 °C. The mixture was then printed, and sintered at 37 °C for 30 mins. The sintered scaffolds were then washed in cold PBS for 45 min and then cultured in media for 7 days. In order to test cell viability and proliferation, we again conducted PrestoBlue cell assays on days 1, 3, 5 and 7 post-fabrication (Fig. 6B). In addition, toluidine blue staining was carried out on day 7 to visualize cells directly on scaffolds (Fig. 6C&D). Cells demonstrated proliferation with increased culture time relative to day 1 (Fig. 6B). Toluidine blue staining showed distribution of the cells along the printed scaffold strand (Fig. 6C), as well as within the strand, shown by breaking and imaging a strand cross-section (Fig. 6D). Cells appeared more densely populated within scaffolds due to them filling large pores inside the material. To fully demonstrate the utility of our system we 3D bioprinted a human-scale implant containing hMSCs (Fig. 7). We modified a STL scan file of a male adult L5 vertebra (Fig. 7A&B); dividing the structure into two halves to allow compatibility with our bioprinting system. We demonstrated the printing of the entire clinically sized implant in <8 mins (4 mins per half implant) (Fig. 7C, Supplementary Video S1), which also incorporated complexity such as overhangs and was several cm^3^ in size (~50 mm height). Implant halves could be bonded with further cell-laden PLGA/PEG paste before sintering (Fig. 7D). Again PF127 gel could be removed by cold washing in PBS. This rapid prototyping of large personalized hard scaffolds, containing hMSCs and a differentiation initiator is the first demonstration of such an approach.

### 3.10 Successful osteogenic hMSC differentiation in bioprinted scaffolds by GET-RUNX2 release

To evaluate the differentiation response of hMSCs co-printed in 3D printed PLGA/PEG scaffolds, we fabricated cell-laden scaffolds incorporating MPs. Both sintering and mechanical properties were similar when MPs were added to the formulation, aligning with previous studies using MP sintering [13,31]. We next carried out differentiation assays with hMSCs by using scaffolds containing GET-RUNX2 MPs (60 μg/ml GET-RUNX2/ml mixture). Cell-laden scaffolds were cultured in OP media, compared to those with blank MPs in OP or OI media, and monitored for 21 days. Differentiation was assessed by OCN staining at week 2, and von Kossa staining for mineralization at week 3 (Fig. S9; black staining is mineralized nodules; white particles the PLGA component). Results were comparable to those achieved without 3D printing (Fig. 4), demonstrating no loss of osteogenic commitment when cells were incorporated and 3D-printed within scaffolds, in comparison with seeding the cells on the scaffolds after sintering. Bone nodules (black staining, Fig. S9B) were observed with GET-RUNX2 which were comparable with that achieved without printing, or when cultured in OI media. This confirms that GET-RUNX2 MP system could be modified for bioprinting and could directly stimulate osteogenic differentiation of hMSCs fabricated within scaffolds.

### 3.11 Regeneration of high density bone by GET-RUNX2 MPs in PLGA/PEG scaffolds *in vivo*

**Fig. 8:**
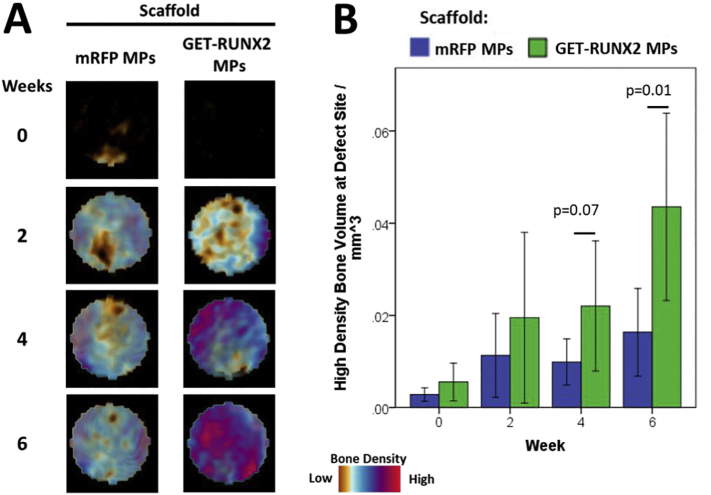
High-density bone regeneration in a drill defect by GET-RUNX2 scaffolds. (A) Representative false colour micro(μ)CT images of the 1 mm diameter drill (250 μm) defect site over a 6 week time period in a mouse implanted with 1- 3 × 104 hMSCs on a 1 mm3 scaffold containing either mRFP MPs (control) or GETRUNX2 MPs. (B) Volume of high density bone detected at defect site (per mm3) calculated as previously [33]. Time indicates the number of weeks after surgery. *n* = 9–12 per group. Graphs show mean with 95% confidence limits with *p*-values indicated.

A femoral drill-hole defect mouse model was used to assess the ability of the PLGA/PEG scaffolds containing GET-RUNX2 MPs to induce bone formation over a 6 week period (Fig. 8, Fig. S10). Human STRO-1+ MSCs were added to scaffold and subsequently used to fill a 1 mm diameter drill-hole defect in the distal femur of athymic mice. *In vivo* CT scans of the defect region were taken at the time of surgery and additionally at 2, 4, and 6 weeks post-surgery in the same animal. The diameter of the analysed defect is 1 mm, centred on the drill hole. The depth of the defect is the thickness of the cortical bone (approx. 250 μm). The images captured were semi-transparent and represent the bone density for the entire 3D reconstruction. This is a better representation of the bone formation rather than an offset thin disc showing the thickness of the defect. The PLGA/PEG scaffold was not visible as the scaffold does not absorb any x-ray energy at the levels used for these scans. Although there was no apparent increase in the total bone volume formed within the defect site (Fig. S10), there was an increase in the volume of high density bone formed in GET-RUNX2 scaffolds at 4 weeks, and a significant increase by 6 week post-surgery (Fig. 8B). The high density definition of bone generated here by GET-RUNX scaffolds is based on the intensity of signal measured by μCT as we have previously published [33] (lowest density bone set at 0.25 g/cm^3^).

## 4 Discussion

GET-RUNX2 releasing PLGA MPs were prepared using S/O/W with high encapsulation efficiency as previously published [13]. We accelerated the protein release by incorporating PBS during the formulation. PBS works as a porogen, increasing MP porosity by generating channels within and on the MP surface [13,31]. Above 90% of the encapsulated P21-RUNX-8R was released in the first 7 day period (Fig. 1E). This release profile was developed to match the previous findings of dose requirements of GET-RUNX2-triggered osteogenic differentiation of hMSCs (*i.e.* two doses of 30 μg/ml in the first week of the study) [9].

The challenging issues in controlled release system are encapsulating and releasing molecules that remain biologically active [47,48]. We have previously shown that GET peptide activity is compromised in the presence of carboxylic acid groups produced upon PLGA degradation. The use of L-Histidine was shown to maintain the activity of GET peptides upon release [13]. Consequently, L-Histidine was added to the formulation with the encapsulated P21-RUNX2-8R. In order to validate the biological functionality for P21-RUNX2-8R post-release, the transduction and subsequent transcriptional activity of the released protein was tested. The results showed that the released P21-RUNX2-8R has activity comparable to the experimental controls, confirming our previous findings and validating the delivery system for targeted delivery of such therapeutic molecules.

Scaffolds were formed by mixing thermo-sensitive PLGA/PEG particles with blank or GET-RUNX2 loaded MPs. PBS was used to create a paste-like material, this was packed in PTFE moulds and incubated for 2 h at 37 °C for sintering. This osteoconductive technology was previously described [26,30] and been demonstrated as 3D printable [27]. However the biomaterial had not been used to program osteogenic differentiation of hMSCs, or employed with GET technology incorporated. The use of this biodegradable polymer along with the GET peptide controlled release delivery system would allow new *in-vivo* applications, especially in bone regeneration. The ability to paste the scaffold at a defect site can offer improved clinical translation when compared to metallic implants [1]. Moreover, this material can be formulated with simple addition of PF127 and Tri-acetin to be 3D printable as demonstrated here; which gives the ability to fill complex and difficult to reach geometric defects or as a pre-fabricated implant [2].

PLGA/PEG scaffolds have potential to be used *in vivo*. Firstly the ability of hMSCs to attach, proliferate and differentiate on scaffolds *in vitro* was assessed. The results showed effective attachment and proliferation of hMSCs on the scaffolds over a 9-day period, confirming cytocompatibility. Next, to prove these scaffolds could host osteogenic differentiation of hMSCs using GET-RUNX2 loaded MPs, we conducted several cell differentiation studies comparing pre-sintered (Fig. 4) and 3D printed systems (Fig. 7). Differentiation markers showed comparable behaviour between scaffolds containing P21-RUNX2-8R loaded MPs and the scaffolds cultured in osteo-inductive (OI) media. Differentiation was comparable between pre-sintered and 3D printed scaffolds (Fig. 4, Fig. 7). Encapsulation of genetically targeted biologics such as TFs in scaffolds should prevent off-target pleiotropic side-effects such as seen with high dose steroid or growth factor stimulation, should reduce the loss by diffusion of such regulatory molecules and will enable safe and effective tissue engineering strategies to be developed [3,4]. The results show that GET-RUNX2 can induce it's own endogenous *RUNX2* expression (Fig. 4, Fig. 7) compared to the samples containing no RUNX2. These findings, here in 3D scaffold structures, are comparable to our previous 2D differentiation findings using GET-RUNX2 to program hMSCs [9]. The use of GET-RUNX2 loaded MPs will help in targeted stem cell reprograming, directly delivering the stimulus needed to induce the expression of osteogenic target genes. This strategy of delivering specific factors for promoting bone formation also helps in preventing undesirable, undirected differentiation which sometimes can see adipose or chondrogenic lineage induction and not osteogenesis from hMSCs, minimizing off-target or systemic side-effects. Our work culminated in demonstrating high-density bone regeneration in a mouse model which shows that the full system can have *in vivo* benefit in a formulation compatible with cells and additive manufacturing. Future work will apply the system to larger pre-clinical animal studies using more complex scaffold implants to confirm the mechanical and biological efficacy at human-sized scales.

## 5 Conclusion

A robust method of fabricating GET-RUNX2-loaded MPs using S/O/W emulsion technique was utilized providing a controlled release system. MPs were mixed with temperature sensitive materials that can solidify at body temperature providing a ‘cancellous bone-like’, mechanically strong scaffold that can fill a bone defect inside the body or generate personalized implants. hMSCs seeded on or within these scaffolds can be induced towards osteogenesis in response to the TF RUNX2 released from the co-formulated MPs. This delivery technology has potential to be valuable in the controlled delivery of various potent therapeutic molecules, including recombinant proteins coupled with GET peptides. We believe that our improved methods to generate hard osteoconductive, 3D bioprinted porous scaffolds that promote hMSC growth and osteogenesis in one combined formulation, has the potential to become a patient-applicable translational technology. Such integrated systems will be vital for new innovative classes of personalized cell-based therapies and regenerative medicine for treating trauma and disease to be exploited.

The following are the supplementary data related to this article.

Supplementary Video S1 3D bioprinting of an L5 adult vertebra (half of structure) Supplementary Video S1

Supplemental Materials and Methods Image 1

Supplemental Figures Image 1

## Author contributions

J.E.D. conceived and initiated the project; H.M.A.A., L.T., M.A. R.O.C·O and J.E.D. designed the experiments; H.M.A.A., L.T., J.M.K., M.A., G.B. S.L. and R.M.H.R. conducted the experiments; R.O.C·O and J.E.D. supervised the study; H.M.A.A, L.T. and J.E.D. wrote the manuscript; All authors approved the final manuscript.

## Declaration of Competing Interest

The authors declare no conflict of interest.
