# Supplementary material for: Genetically-programmed, mesenchymal stromal cell-laden & mechanically strong 3D bioprinted scaffolds for bone repair
Source: J Control Release. 2020 Sep 10;325:335–46. doi: 10.1016/j.jconrel.2020.06.035 (PMC7445425; doi:10.1016/j.jconrel.2020.06.035)
Supplement: Supplementary file 2 — Supplemental Materials and Methods [file mmc2.pdf]

Supplemental Material and Methods

1. MP characterization: size and morphology

A thin layer of MPs were placed onto an adhesive stub and gold coated using a SCD030 gold sputter coater (Balzers Union Ltd., Lichtenstein), for five minutes at 30 mA. The morphology of the MPs was assessed by scanning electron microscopy (SEM), using a JEOL 6060L imaging system (JEOL Ltd., Hertfordshire, UK) with the accelerating voltage set to 10 kV.

The mean MP diameter and size distribution were measured using a Coulter LS230 particle size analyser (Beckman, UK). MPs were suspended in HPLC grade water, under stirring conditions. Size distribution data was collected after achieving an obscuration value of 8–12%.

2. MP characterization: Determination of protein loading efficiency

Measurement of protein content in the MPs was conducted as previously described [1-3]. Ten milligrams of PLGA MPs was added to 750µl DMSO and shaken until completely dissolved, 2150µl of 0.02% (w/v) SDS in 0.2 M NaOH was then added and incubated at room temperature until a clear solution was obtained. The Micro BCA protein assay kit was used to measure the total protein content and compared against a freshly generated standard curve of P21-RUNX2-8R. One hundred and fifty microlitre sample volume was mixed with 150µL Micro BCA working reagent and incubated at 37 ºC for 2 h. Absorbance at 562 nm was then measured using a plate reader (Infinite M200, Tecan UK Ltd.).

3. MP characterization: P21-RUNX2-8R release

MPs were suspended in 1 ml Growth media (GM) or HBSS. Samples were rocked on a 3D shaker (Gyrotwister, Fisher Scientific UK Ltd) at 5 rpm in a humidified incubator at 37 ºC. At defined time points, samples were centrifuged and the supernatant was removed from the MPs and replaced with equal volume of fresh GM or HBSS. The supernatants were assayed for protein content using Micro BCA assay kit (HBSS samples). Released protein contents were compared against freshly generated standard curve of P21-RUNX2-8R.

4. Cell culture

Human Mesenchymal Stem Cells (hMSCs) were purchased from Lonza (21 year old male donor) and were maintained in growth medium (MSCGM™ Mesenchymal Stem Cell Growth Medium BulletKit™, Lonza) in 5% (v/v) CO_2 humidified incubator at 37ºC. hMSCs were sub-cultured at 80% confluence preventing spontaneous differentiation and contact inhibition of growth. hMSCs were used between passage 4 and 6 for all experiments. For 3D printing we used immortalized hMSCs (iHMSCs) as previously described [4]. Unless stated otherwise, all experiments were carried out thrice with triplicates.

5. GET peptide transduction activity

Forty thousand cells/well (in 24-well plates) were seeded; incubated for 6 hours for attachment. Culture media was then replaced with the media containing the released P21-RUNX2-8R-FITC protein (protein released from MPs, section 2.5). After overnight transduction, cells were washed with PBS, trypsinized with Trypsin-EDTA (Lonza) and fixed in 4% (w/v) PFA (Sigma) for flow cytometry.

6. Luciferase reporter assay

Cells were transfected with firefly luciferase reporter (kindly gifted by Dr Haijun Zhang, Indiana University) mOG2-Luc along with an internal control, *Renilla* luciferase reporter pRL-TK as previously described [5]. hMSCs were transduced with the P21-RUNX2-8R proteins released from MPs (Section 2.5) before and after transfection to achieve highest induction possible as previously noted [7]. As a positive control to compare the promoter activity, hMSCs were transfected with pSIN-RUNX2 plasmid DNA (1 µg) [6] using Lipofectamine 2000 (Invitrogen, Paisley, UK) and analyzed for luciferase activity. Fresh P21-RUNX2-8R equivalent to the quantity of protein released from MPs was transduced in fresh wells of cells to compare the activity of the released protein verses fresh protein at the same concentration. Relative luciferase activities were measured using dual luciferase assay kit (Promega).

7. Flow cytometry and microscopy

Fixed cells were analysed on a Beckman Coulter Astrios EQ Flow Cytometer using 488 laser. (20,000 cells; gated on single cells by forward/side scatter). Mean fluorescence was used for statistical analyses with background from un-transduced cells subtracted and values were then calculated in ratio to the experimental control. For fluorescence imaging, cells were imaged with fluorescence microscope using a FITC filter (Leica DM IRB).

8. PLGA/PEG particle production

Particles were fabricated from blends of poly(DL-lactic acid-co-glycolic acid) (PLGA) 85:15 53 kDa (Lakeshore Biomaterials, USA) and poly(ethylene glycol) 400 (PEG)

(Sigma Aldrich, UK) as previously described [7]. Briefly, a mixture of 93.5%:6.5% PLGA:PEG (w/v) was blended at 80–90°C, mixed and allowed to cool. Cooled polymer was ground into particles and sieved to obtain the 50–100 μm size fraction.

9. Scaffold preparation

Scaffolds were prepared in PTFE moulds producing cylindrical scaffolds of 3 mm length and 6 mm diameter. PLGA/PEG particles were mixed with P21-RUNX2-8R loaded MPs (or blank MPs for blank scaffolds). The required quantity of MPs and PLGA/PEG particles for each scaffold was individually weighed and mixed. The mixture was then mixed with either PBS at a ratio of 1:0.6 (mixture: PBS; w/v), PF127 18% at 1:1.5 ratio (mixture: PF127) or with 1% Tri-acetin with 18% PF127 at 1:1.5 ratio (mixture: Tri-acetin/PF127; w/v). The paste was packed into the mould which was placed at 37°C for 2 hours or 30 minutes (for samples containing Tri-acetin) to allow scaffold stabilization.

10. Scaffold mechanical characterization

Compressive strength and Young's modulus was assessed as previously published by Rahman et al. (2013) [8]. Readings were taken in triplicate of conventional or PF127-containing extruded scaffolds using a TA.HD+ texture analyzer (Stable Microsystems) with a Peltier unit heated to 37°C and a scaffold contact area of 28.26 mm^2. Scaffolds were placed or extruded into moulds and removed after sintering, washed as detailed and immediately tested on the texture analyser without drying. A 50 kg (490 N) load cell, and a P/10 probe with 0.04 mm/s test speed was used. Strain was set at 30% with a 5g trigger force. The compressive strength was the maximum stress at the yield point of the sample (the fracture peak). The stress–strain curve

generated was used to calculate the Young's modulus (elastic modulus), as a measure of the scaffold stiffness. The elastic modulus was calculated by determining the slope of the stress–strain curve along the elastic portion of deformation.

11. Alkaline phosphatase (ALP) assay

ALP expression was measured at day 7 using a SigmaFAST pNPP kit. Scaffolds were transferred to new 24 well plate and pNPP solution, made to manufacturer's guidelines, was added (500µl) to the wells and incubated at 37ºC for 45 minutes. Absorbance at 405 nm was then measured (three readings per well) using Tecan plate reader.

12. OSTEOCALCIN immunostaining

After 14 days, scaffolds were rinsed with PBS, fixed in 4% (w/v) PFA (Sigma) in deionized water for 20 min, stained with anti-OSTEOCALCIN(OCN) antibody (Millipore; AB10911; 1:200), detected with secondary antibody Alexa Fluor 546 (Abcam, Cambridge, UK) and imaged using the confocal unit LSM 780 of the Zeiss Elyra PS1 microscope.

13. von Kossa staining

After 21 days, scaffolds were rinsed with deionized water, fixed with 4% (w/v) PFA for 20 minutes. Fixed scaffolds were incubated with 1% silver nitrate solution (Sigma, UK) under exposure to UV light for 60 minutes. Scaffolds were washed with deionized water and then with 2.5% sodium thiosulfate solution (Sigma, UK) for 3 times, followed by deionized water. Mineralized nodules were visualized as black deposits using a Nikon SMZ1500 dissection microscope.

14. Gene expression analysis

The scaffolds with hMSCs were homogenized with RLT buffer (RNAeasy kit) using a hand-held homogenizer and total RNA was extracted using QIAShredder (Qiagen) and RNAeasy kit (Qiagen) according to the manufacturer's instructions. RNA samples were treated with DNase I (Invitrogen, UK), quantified using a NanoDrop 1000 spectrophotometer (Thermo-Fisher, UK). Expression of osteogenic genes (*RUNX2* – Hs00231692_m; *OSX/SP7* – Hs00541729_m1; *OPN* – Hs00959010_m1) were determined relative to *ACTB* - Hs99999903-m1 according to the TaqMan gene expression assay protocol (Applied Biosystems/ Life Technologies). TaqMan primers and probes were from Applied Biosystems. All TaqMan PCR reactions were performed in triplicates with three biological repeats.

15. Scaffold µCT

Samples were analysed using Skyscan 1172 (Skyscan, Belgium) desktop x-ray CT scanner with a pixel resolution of 12.2 µm, x-ray source current 800 µA and voltage 50 kV. Samples were mounted vertically and rotated thorough 360°. Captured images were then reconstructed using the NRecon software (Skyscan, Belgium). The construct was then analysed with 3D histomorphometric using the CTAn software by thresholding the sample lower grey 20 and upper grey 150. For the reconstruction of the cell-seeded samples, cells were stained with osmium tetroxide and the previous method was used to distinguish the cells from the 3D pictures [7] . The thresholds for the cells were lower grey 155 and upper grey 255. Materialise 3-matic software (version 9) was used for image overlays.

16. References

[1] G.T.S. Kirby, L.J. White, C.V. Rahman, H.C. Cox, O. Qutachi, F.R.A.J. Rose, D.W. Hutmacher, K.M. Shakesheff, M.A. Woodruff, PLGA-Based Microparticles for the Sustained Release of BMP-2, Polymers 3(1) (2011) 571.

[2] L.J. White, G.T.S. Kirby, H.C. Cox, R. Qodratnama, O. Qutachi, F.R.A.J. Rose, K.M. Shakesheff, Accelerating protein release from microparticles for regenerative medicine applications, Materials Science and Engineering: C 33(5) (2013) 2578-2583.

[3] H.A.-D.M. Abu-Awwad, L. Thiagarajan, J.E. Dixon, Controlled release of GAG-binding enhanced transduction (GET) peptides for sustained and highly efficient intracellular delivery, Acta Biomaterialia 57 (2017) 225-237.

[4] L. Thiagarajan, H.A.M. Abu-Awwad, J.E. Dixon, Osteogenic Programming of Human Mesenchymal Stem Cells with Highly Efficient Intracellular Delivery of RUNX2, Stem cells translational medicine 6(12) (2017) 2146-2159.

[5] H. Zhang, Y. Pan, L. Zheng, C. Choe, B. Lindgren, E.D. Jensen, J.J. Westendorf, L. Cheng, H. Huang, FOXO1 Inhibits Runx2 Transcriptional Activity and Prostate Cancer Cell Migration and Invasion, Cancer Research (2011).

[6] J.E. Dixon, E. Dick, D. Rajamohan, K.M. Shakesheff, C. Denning, Directed Differentiation of Human Embryonic Stem Cells to Interrogate the Cardiac Gene Regulatory Network, Molecular Therapy 19(9) 1695-1703.

[7] A. Dhillon, P. Schneider, G. Kuhn, Y. Reinwald, L.J. White, A. Levchuk, F.R. Rose, R. Muller, K.M. Shakesheff, C.V. Rahman, Analysis of sintered polymer scaffolds using concomitant synchrotron computed tomography and in situ mechanical testing, Journal of materials science. Materials in medicine 22(12) (2011) 2599-605.

[8] C.V. Rahman, G. Kuhn, L.J. White, G.T. Kirby, O.P. Varghese, J.S. McLaren, H.C. Cox, F.R. Rose, R. Muller, J. Hilborn, K.M. Shakesheff, PLGA/PEG-hydrogel composite scaffolds with controllable mechanical properties, J Biomed Mater Res B Appl Biomater 101(4) (2013) 648-55.
