# Supplementary material for: Genetically-programmed, mesenchymal stromal cell-laden & mechanically strong 3D bioprinted scaffolds for bone repair
Source: J Control Release. 2020 Sep 10;325:335–46. doi: 10.1016/j.jconrel.2020.06.035 (PMC7445425; doi:10.1016/j.jconrel.2020.06.035)
Supplement: Supplementary file 3 — Supplemental Figures [file mmc3.pdf]

Figure S1.

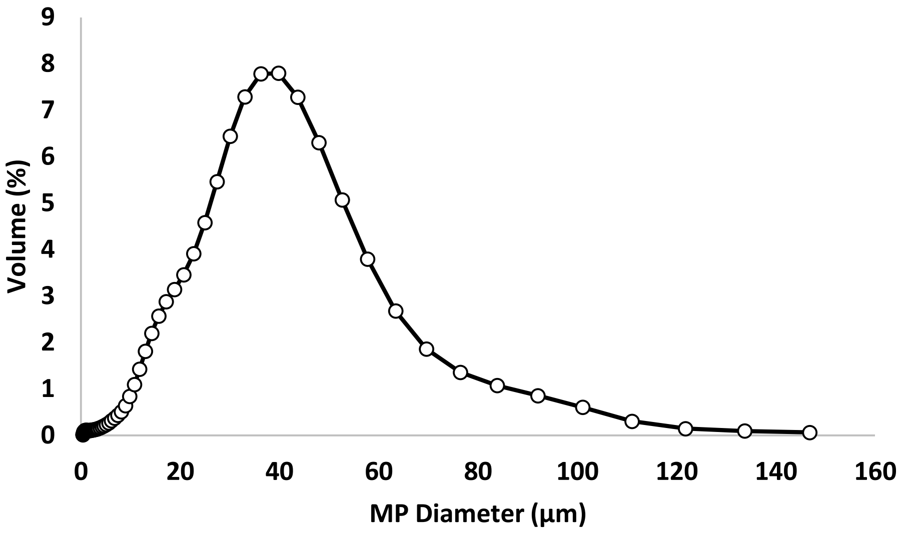

Figure S1. Size distribution of GET-RUNX2 PLGA MPs. Representative size distribution of GET-RUNX2-loaded PLGA MPs generated with 0.1% (w/w) PBS, 25% (w/w) L-Histidine.

Figure S2.

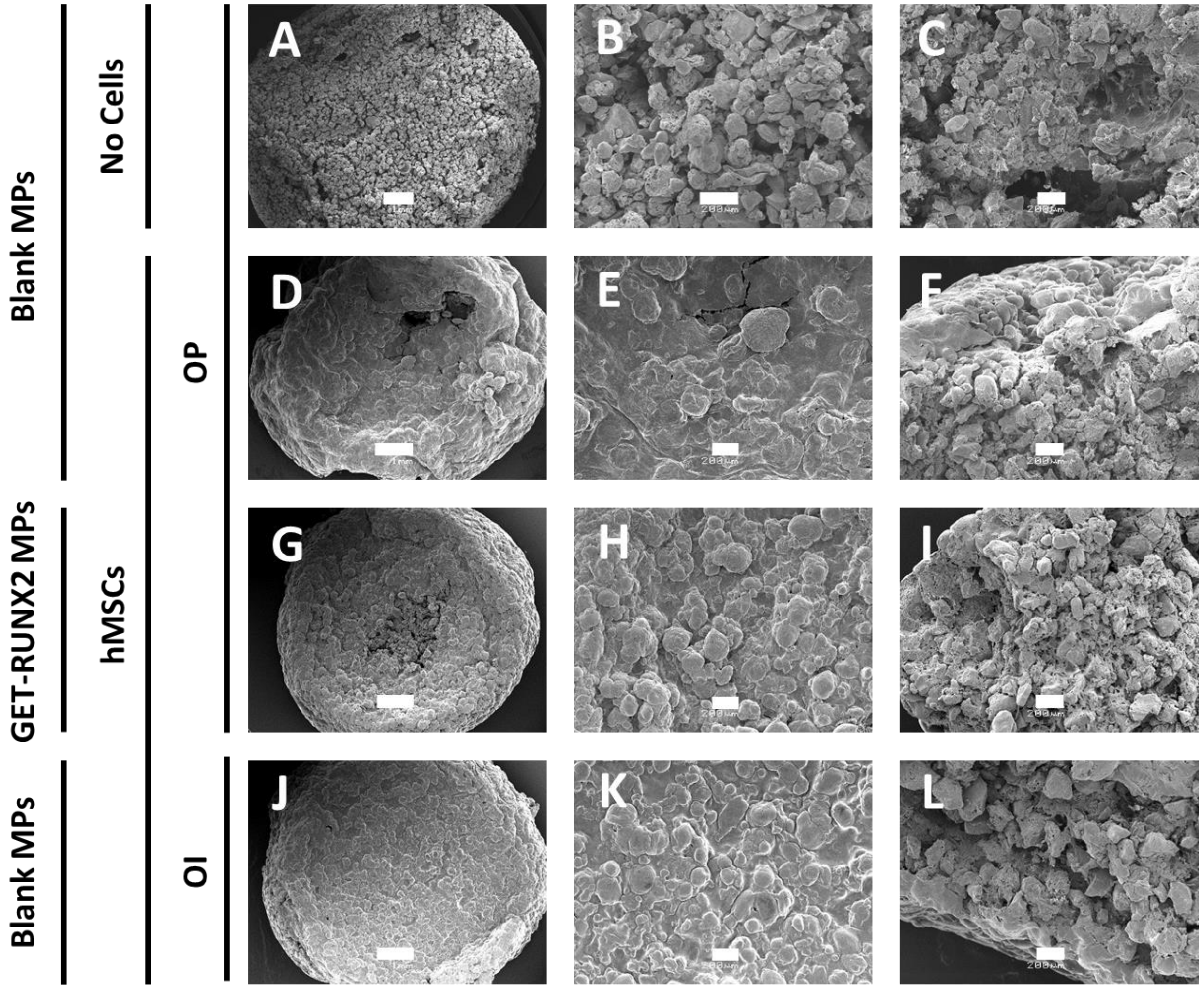

Figure S2. Morphology of PLGA/PEG scaffolds after prolonged hMSC culture using SEM. SEM images of scaffolds with and without hMSCs cultured in growth media for 21 days. No cells represents scaffold prepared from blank MPs without cells kept in osteo-permissive (OP) media. Blank MPs represents cell-seeded scaffold containing blank MPs cultured in osteo-permissive (OP) media. RUNX2-loaded MPs represents cell-seeded scaffolds containing P21-RUNX2-8R loaded MPs cultured in osteo-permissive (OP) media. OI media represents cell seeded scaffolds containing blank MPs cultured in osteo-inductive (OI) media. C, F, I and L are for scaffolds cut in half to show internal morphology. Scale bars for A, D, G, J are 1mm; B, E, H, K are 200µm; and C, F, I, L are 200µm, respectively.

Figure S3.

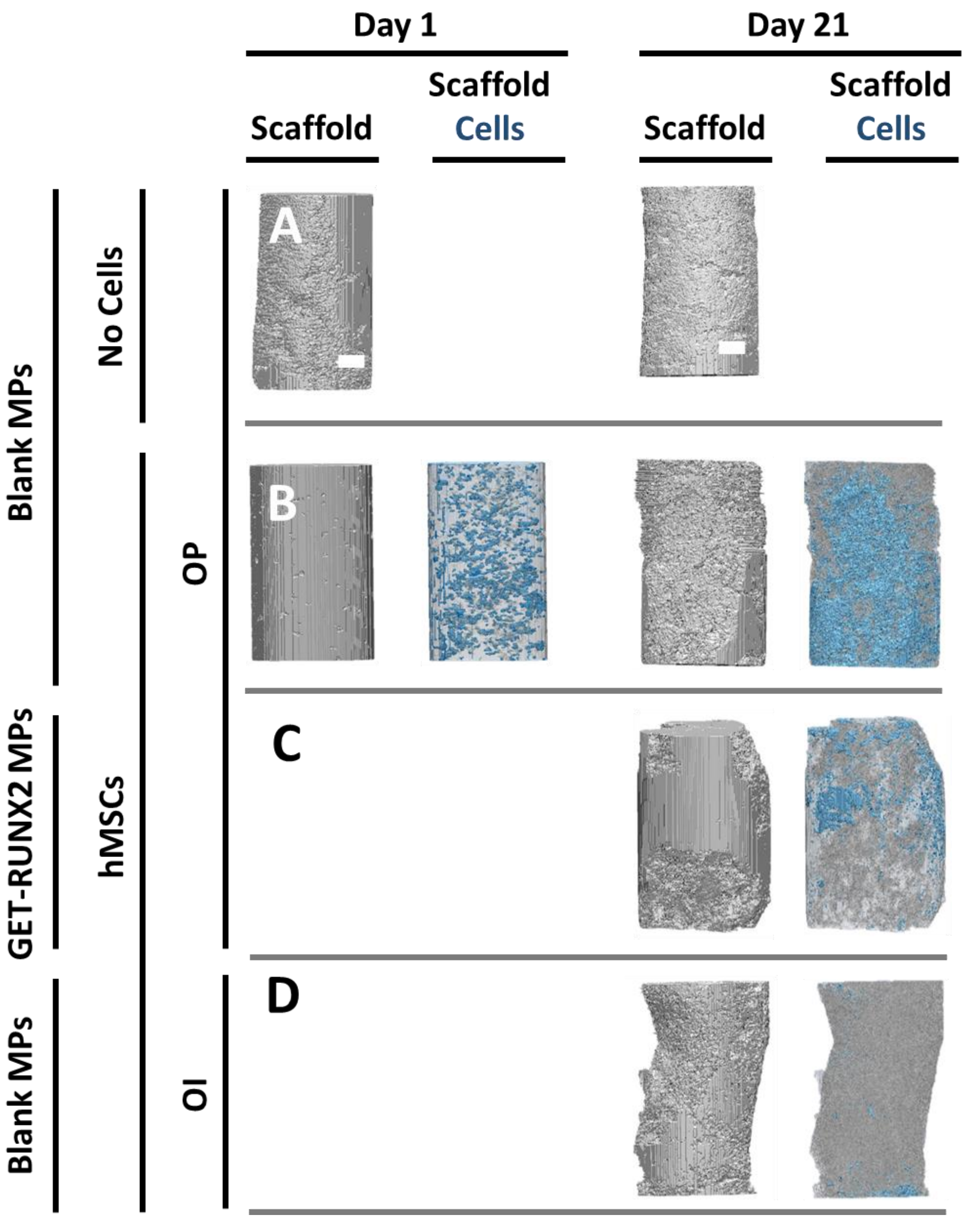

Figure S3. Microstructural analysis of PLGA/PEG scaffolds. Micro(µ)CT imaging for scaffolds cultured in different conditions. Scaffolds were treated with osmium tetroxide prior to scan. (A) Non-seeded scaffold prepared from blank MPs placed in cell culture media at day 1 and day 21. (B) Cell seeded scaffold prepared from blank MPs cultured in osteo-permissive media for 21 days (left image) and merged cells and scaffold showing cell distribution (right). (C) hMSC seeded scaffold prepared from P21-RUNX2-8R loaded MPs cultured in osteo-permissive media (OP) for 21 days (left image) and merged cells and scaffold showing cell distribution (right). (D) hMSC seeded scaffold prepared with blank MPs cultured in osteo-inductive (OI) media for 21 days (left image) and merged cells and scaffold showing cell distribution (right). Cell-seeded scaffold at day 1 in all conditions are shown, scaffold (left image) and merged cells and scaffold showing cell distribution (right). Grey is scaffold; blue is osmium-stained cells. Scale bar is 1mm.

Figure S4.

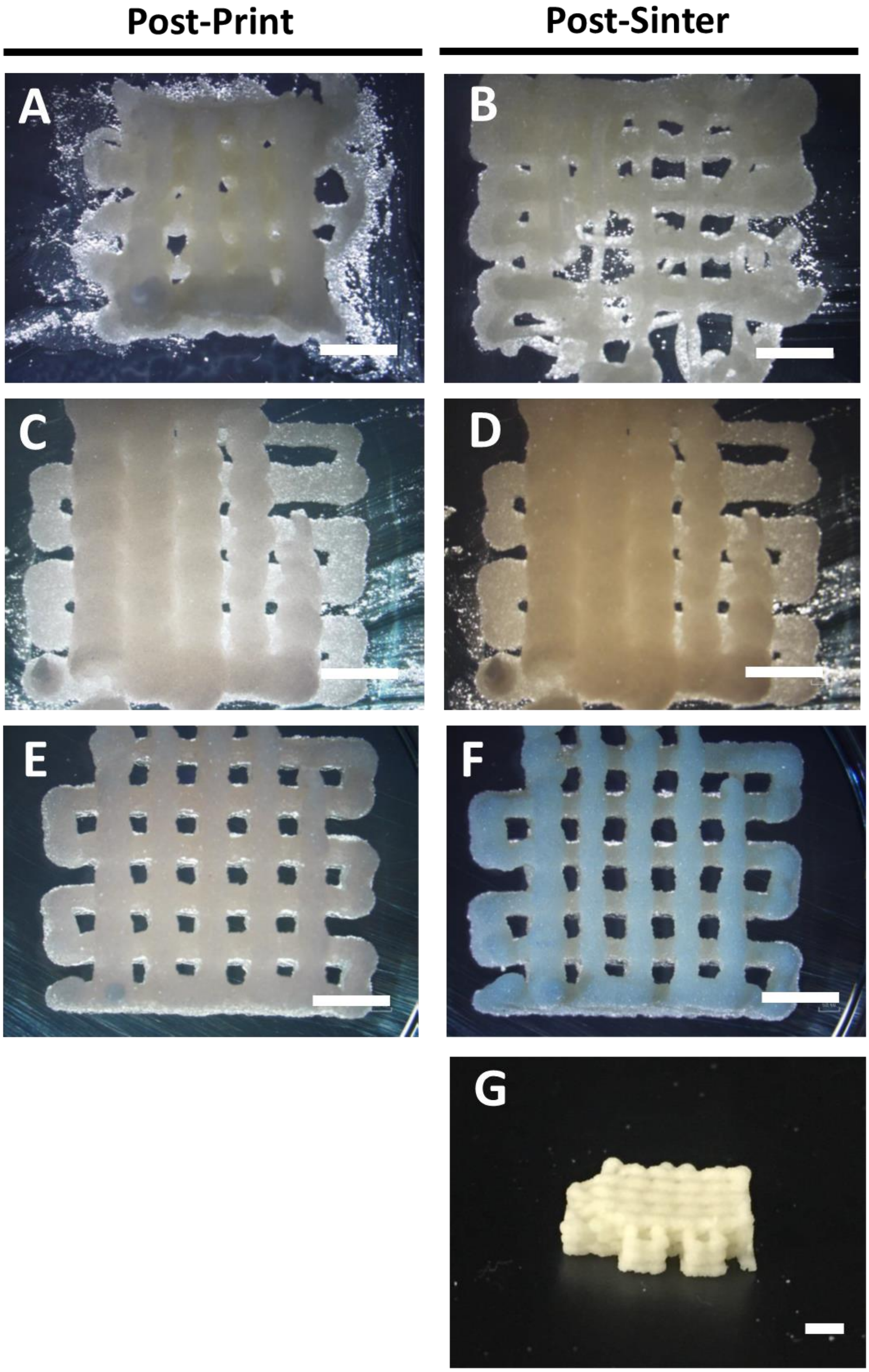

Figure S4. PLGA/PEG pastes can be 3D printed with addition of Pluronic F127. Various ratios and concentrations of Pluronic F127 (PF127) were utilized to 3D print PLGA/PEG particles. Dissection microscope images (A, B) Post-print and post-sinter of PLGA/PEG: PF127 15% (1:1.2 ratio). (C, D) Post-print and post-sinter of PLGA/PEG: PF127 15% (1:1.3 ratio). (E-G) Post-print and post-sinter of PLGA/PEG: PF127 18% (1:1.5 ratio). (G) Shows multi-layered sintered scaffold. Sintering was for 2 hours at 37°C. Scale bars represents 1mm.

Figure S5.

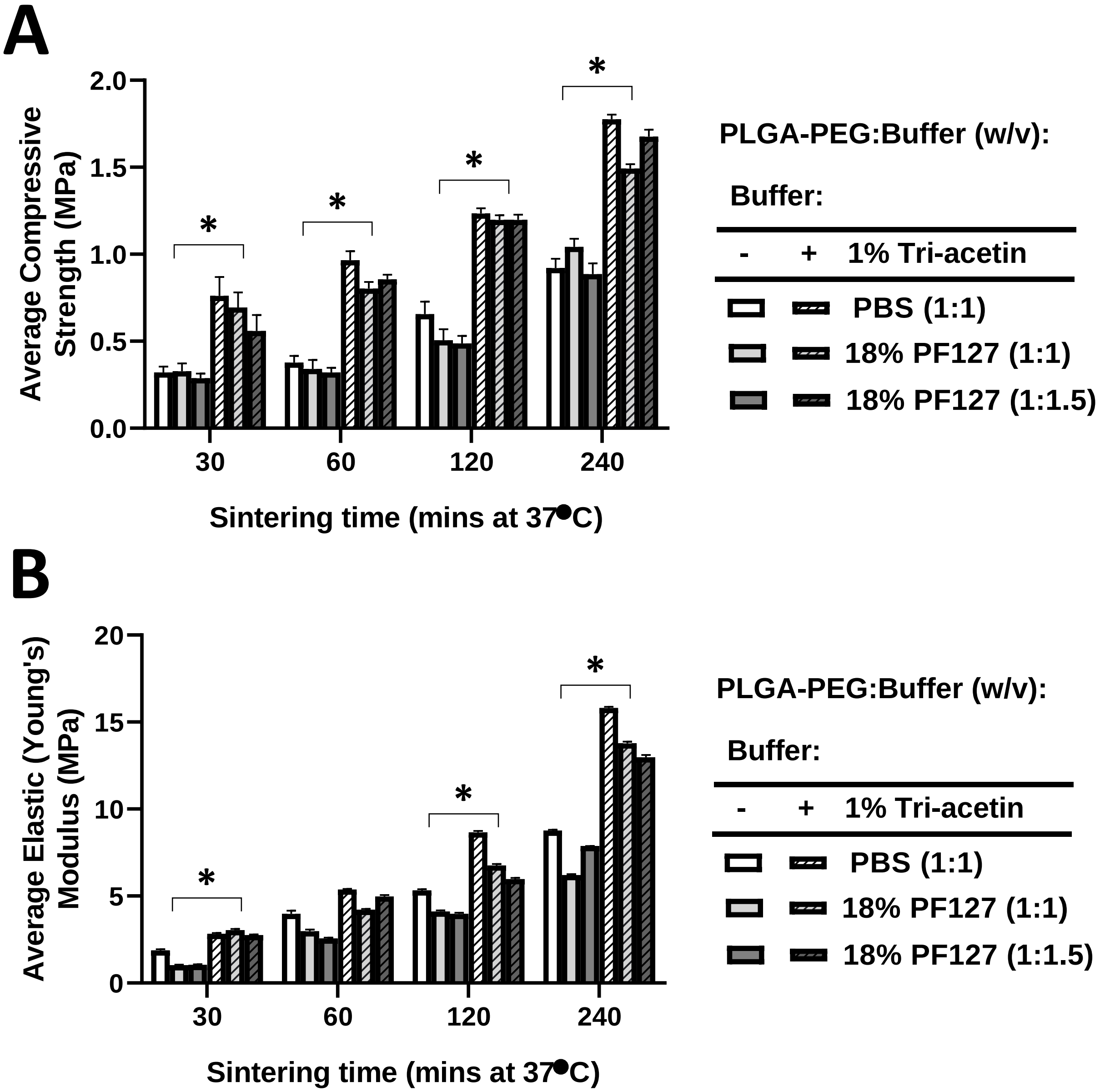

Figure S5. Enhanced sintering and mechanical properties of PLGA/PEG scaffolds with Tri-acetin. (A) Compressive strength (MPa), and (B) Elastic (Young's) modulus (MPa) of PLGA-PEG:PBS (1:1) or PLGA-PEG:18% PF127 scaffolds (1:1 or 1:1.5) with and without 1% (v/v) Tri-acetin (w/v) sintered for 30 to 240 min at 37°C. Scaffolds were tested directly after sintering. Error bars indicate SD; n=6. * $p<0.05$ comparing with and without Tri-acetin.

Figure S6.

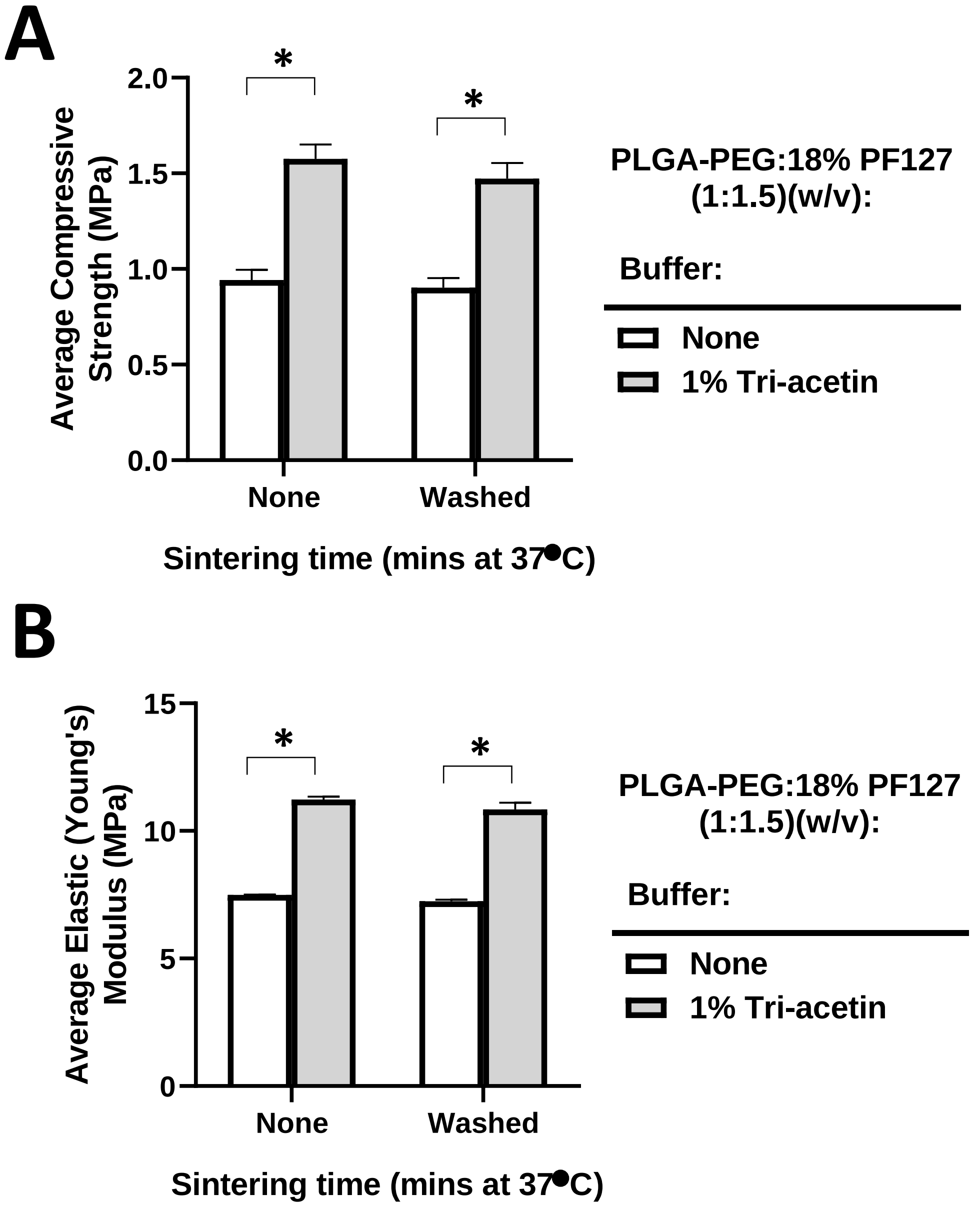

Figure S6. Cold washing does not affect mechanical properties of PLGA/PEG scaffolds. (A) Compressive strength (MPa), and (B) Elastic (Young's) modulus (MPa) of PLGA-PEG: 18% PF127 scaffolds with and without 1% (v/v) Tri-acetin (1:1.5) (w/v) sintered for 240 min at 37°C. Scaffolds were tested after sintering or cold PBS washed at 4°C for 45mins before testing. Error bars indicate SD; n=6. * $p<0.05$.

Figure S7.

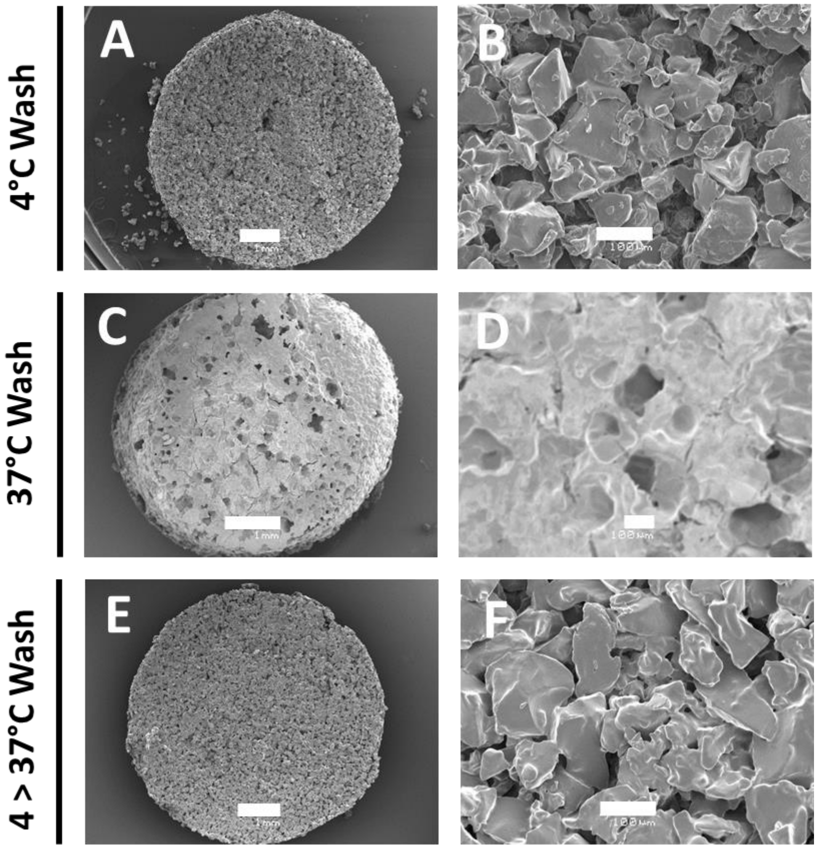

Figure S7. Cold washing removes PF127 from PLGA/PEG scaffolds. SEM analysis of scaffold morphology. PLGA/PEG mixed with 1% Tri-actin and 18% PF127 (1:1.5 ratio) scaffolds were sintered at 37°C. Scaffolds were then washed with PBS at 4°C (A,B), 37°C (C,D) or 4°C then at 37°C (E,F) for 45mins. Scale bars for A, C, E and B, D, F are 1mm and 100µm, respectively.

Figure S8.

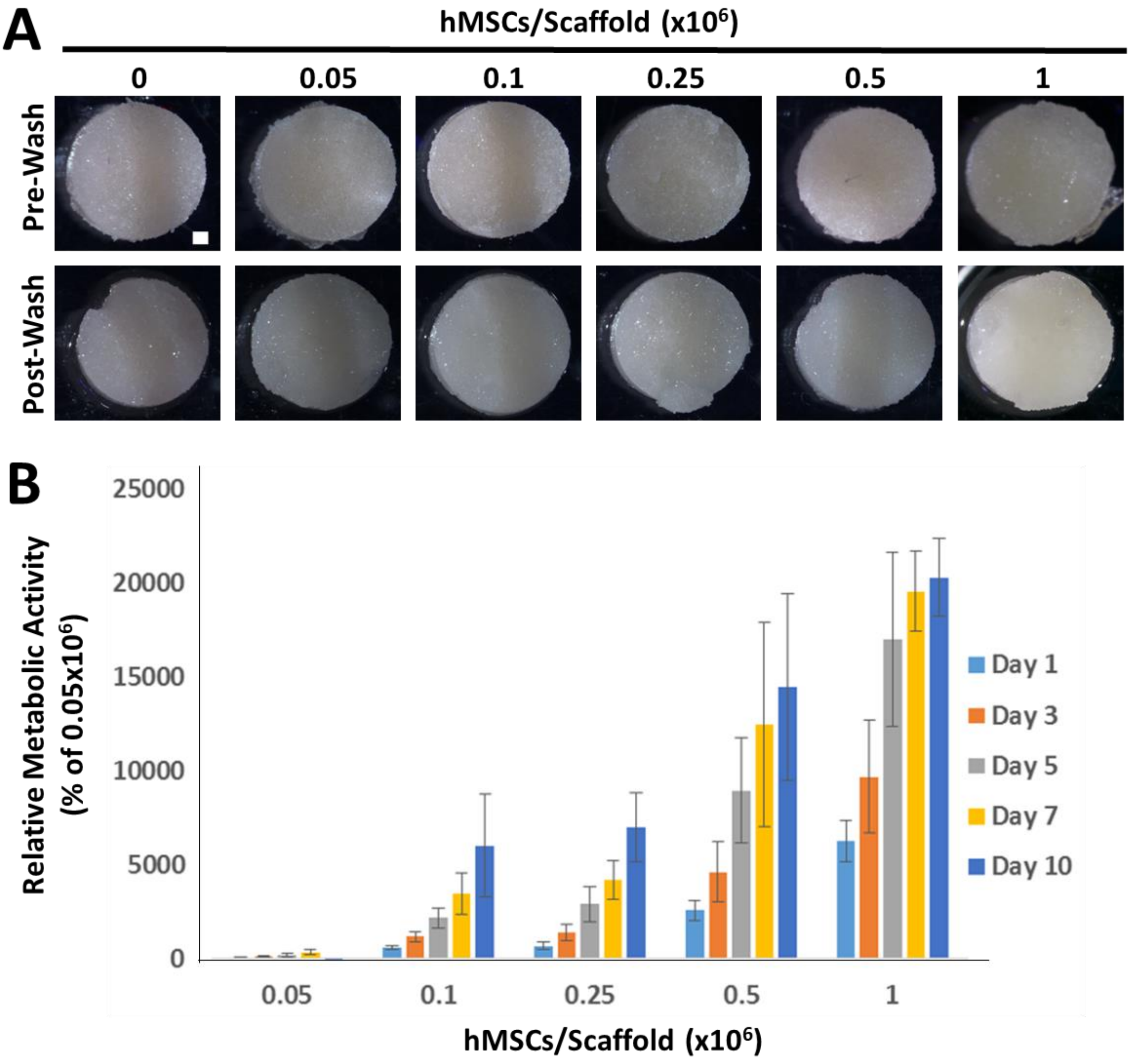

Figure S8. Scaffold integrity and hMSC proliferation after removal of PF127. Various densities of cells (0-1x10^6 ihMSCs/ml) were mixed with 1% Tri-acetin, 18% PF127 (w/v) solution and added to PLGA/PEG forming a paste at room temperature. The paste was then loaded into Teflon mould to produce cylindrical scaffolds (6mm X 3mm). The scaffolds were then sintered at 37°C for 2 hours. The scaffolds were then washed in PBS at 4°C for 45 minutes, transferred to 24 well plate and immersed in 1 ml media before culturing at 37°C for the entire study. (A) Microscope images for scaffolds after sintering and after washing showing intact scaffolds formulated with different cell numbers. Scale bar is 500µm. (B) PrestoBlue cell assay at different time points assessing cell retention, viability and proliferation in the cultured scaffolds. Error bars indicate SD (n = 3). Data is presented as a % of the 0.05x10^6 hMSC/ml sample at day 1.

Figure S9.

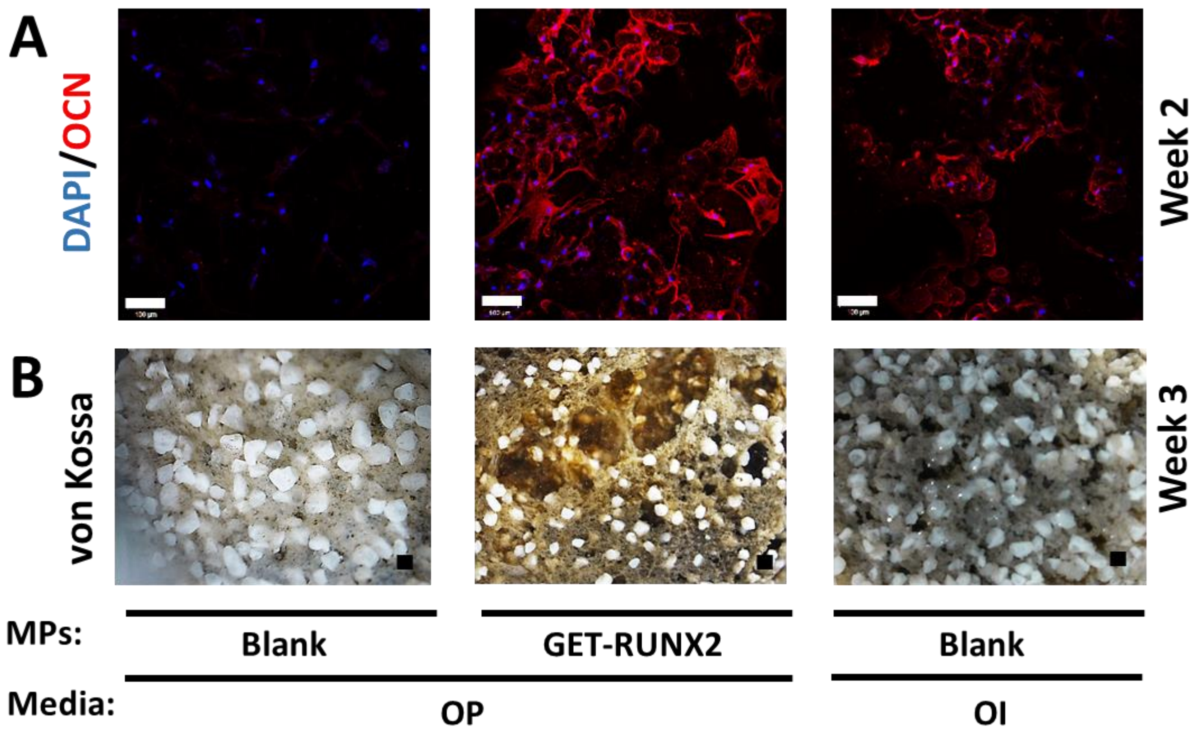

Figure S9. hMSC osteogenic differentiation in 3D bioprinted PLGA/PEG scaffolds. Differentiation markers for hMSCs 3D bioprinted within scaffolds in osteo-permissive (OP) and osteo-inductive media (OI). Scaffolds were prepared by mixing PLGA/PEG particles with blank or GET-RUNX2 loaded MPs, and 1% Tri-acetin, 18% PF127 in 1:1.5 ratio (total particles : PF127). (A) OSTEOCALCIN (OCN) immunostaining (red) with DAPI (blue) at week 2 (scale bar 100 µm). (B) Von kossa-stained samples at week 3 (scale bar 100 µm). Blank MPs represents cell seeded scaffold containing blank MPs cultured in osteo-permissive (OP) media. Loaded MPs represents cell-seeded scaffolds containing P21-RUNX2-8R-loaded MPs cultured in osteo-permissive (OP) media. OI media represents cell seeded scaffolds containing blank MPs cultured in osteo-inductive media (OI).

Figure S10.

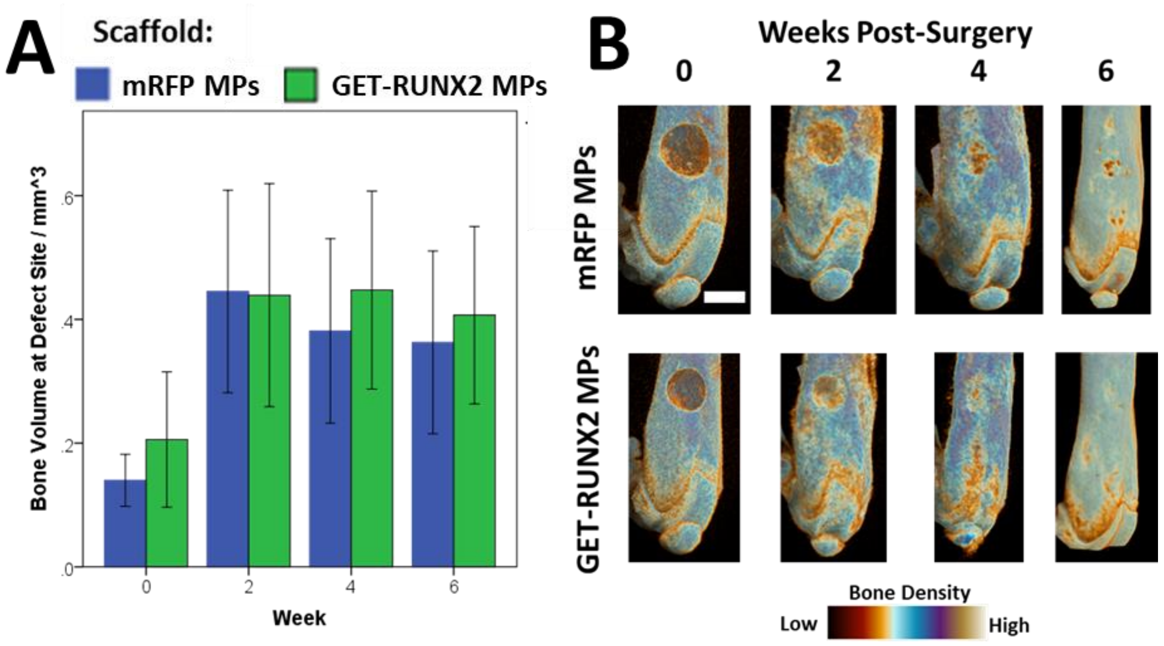

Figure S10. Similar bone volume at defect site using PLGA/PEG scaffolds with control (mRFP) or GET-RUNX2 MPs. (A) Total volume of bone detected at the defect site over a 6 week time period in mice implanted with 1-3x10^4 hMSCs on a 1mm^3 scaffold containing either GET-mRFP MPs (control) or GET-RUNX2 MPs. (B) Representative false colour CT images showing drill defect area. Time indicates the number of weeks after surgery. n=9-12 per group. Graphs show mean with 95% confidence limits.
